# Revealing the heterogeneity of treatment resistance in less‐defined subtype diffuse large B cell lymphoma patients by integrating programmed cell death patterns and liquid biopsy

**DOI:** 10.1002/ctm2.70150

**Published:** 2024-12-27

**Authors:** Wei Hua, Jie Liu, Yue Li, Hua Yin, Hao‐Rui Shen, Jia‐Zhu Wu, Yi‐Lin Kong, Bi‐Hui Pan, Jun‐Heng Liang, Li Wang, Jian‐Yong Li, Rui Gao, Jin‐Hua Liang, Wei Xu

**Affiliations:** ^1^ Department of Hematology The First Affiliated Hospital of Nanjing Medical University, Jiangsu Province Hospital Nanjing China; ^2^ Department of Hematology The Third Affiliated Hospital of Nanjing Medical University Nanjing China; ^3^ Department of Medical Affairs Nanjing Geneseeq Technology Inc Nanjing China; ^4^ Department of Endocrinology The First Affiliated Hospital of Nanjing Medical University, Jiangsu Province Hospital Nanjing China

**Keywords:** ctDNA, DLBCL, liquid biopsy, lymphoma microenvironment, machine learning, prognostic nomogram, programmed cell death

## Abstract

Precision medicine in less‐defined subtype diffuse large B‐cell lymphoma (DLBCL) remains a challenge due to the heterogeneous nature of the disease. Programmed cell death (PCD) pathways are crucial in the advancement of lymphoma and serve as significant prognostic markers for individuals afflicted with lymphoid cancers. To identify robust prognostic biomarkers that can guide personalized management for less‐defined subtype DLBCL patients, we integrated multi‐omics data derived from 339 standard R‐CHOP‐treated patients diagnosed with less‐defined subtype DLBCL from three independent cohorts. By employing various machine learning algorithms, we pinpointed eight pivotal genes linked to PCD, specifically FLT3, SORL1, CD8A, BCL2L1, COL13A1, MPG, DYRK2 and CAMK2B. Following this, we established a Programmed Cell Death Index (PCDI) utilizing the aforementioned genes and amalgamated it with pertinent clinical characteristics to formulate a predictive nomogram model for prognosis. We observed a significant correlation between the PCDI, pre‐treatment circulating tumour DNA (ctDNA) burden, minimal residual disease (MRD) status and immune features. Furthermore, our research indicated that patients with elevated PCDI scores could potentially show resistance to conventional chemotherapy treatments, yet they might derive an advantage from alternative inhibitors targeting specific signalling pathways. Conclusively, leveraging these results, we have created an online analytical tool (https://xulymphoma.shinyapps.io/PCDI_pred/) designed for the prognostic prediction of patients with less‐defined subtype DLBCL. This tool facilitates the forecasting of outcomes for these patients, enhancing the precision of their clinical management.

**Key points:**

- Developing the Programmed Cell Death Index (PCDI) utilizing multiple machine learning algorithms for patients with less‐defined subtype diffuse large B‐cell lymphoma.
- The difference in clinical characteristics, circulating tumour DNA burden and immune profiling between patients with distinct PCDI groups.
- A potentially effective regimen was speculated for patients with high PCDI scores who tend to exhibit worse progression‐free survival.

## Graphical abstract

1. Developing the Programmed Cell Death Index (PCDI) utilizing multiple machine learning algorithms for patients with less‐defined subtype diffuse large B‐cell lymphoma.

2. The difference in clinical characteristics, circulating tumour DNA burden and immune profiling between patients with distinct PCDI groups.

3. A potentially effective regimen was speculated for patients with high PCDI scores who tend to exhibit worse progression‐free survival.

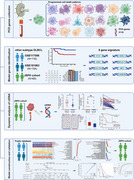

## 1 INTRODUCTION

Among molecular subtypes developed by Staudt et al. in 2020,^1^ approximately 50% diffuse large B‐cell lymphoma (DLBCL) remain unclassified due to the lack of characteristic genetic mutations. Patients diagnosed with less‐defined subtype DLBCL exhibit great heterogeneity within the response to first‐line standard treatment, leading to considerable variation in overall survival (OS) and progression‐free survival (PFS).^2^ In the GUIDANCE‐01 trial, the rituximab, cyclophosphamide, doxorubicin, vincristine, and prednisone (R‐CHOP)+X regimen showed almost no difference in complete response rate (CRR) and OS compared to the control group among less‐defined subtype DLBCL patients.^3^ Hence, there is a pressing requirement for additional research to identify biomarkers capable of forecasting the effectiveness of targeted and immunotherapies for these patients.

Programmed cell death (PCD), a distinct type of cell demise, is governed by a suite of biomacromolecules.^4^, ^5^ When the intricate regulation of PCD metabolic pathways is disrupted, it can lead to the buildup of genetically impaired or abnormal cells. This accumulation fosters their continuous and unchecked growth, culminating in detrimental health consequences.^6^ Although the roles of different PCD patterns in DLBCL have been reported, the intricate interplay between the 19 distinct forms of PCD and the immune response against cancer in less‐defined subtype DLBCL is not yet fully understood.

In this study, 270 patients from multiple public databases and 69 from the Jiangsu Province Hospital (JSPH) cohort, all diagnosed with less‐defined subtype DLBCL, were enrolled in this analysis. We have pinpointed eight genes that are linked to PCD in the less‐defined subtype DLBCL. Following this discovery, we constructed a Programmed Cell Death Index (PCDI) to investigate the correlation among these key genes, the PCDI itself, ctDNA burden, other clinical characteristics and the disease progression of DLBCL patients classified as less‐defined subtype. Furthermore, we conducted a thorough characterization of the genetic and mutational profiles across various risk groups stratified by PCDI scores. This comprehensive analysis allowed us to devise a prognostic model capable of precisely forecasting survival outcomes for patients with DLBCL classified as a less‐defined subtype. Additionally, we delved into the complex interactions between the model genes, PCDI scores, and the lymphoma microenvironment (LME) to better understand their collective influence on disease progression and treatment response.

## 2 METHODS AND MATERIALS

### 2.1 Clinical and genetic data

In this research, we conducted an examination of both clinical and genetic data derived from three distinct cohorts of patients diagnosed with DLBCL. The first cohort (JSPH cohort) included 69 newly diagnosed less‐defined subtype DLBCL patients diagnosed in the First Affiliated Hospital of Nanjing Medical University (Jiangsu Province Hospital) from April 2021 to September 2023 (Table 1). The first‐line treatments of all the newly diagnosed DLBCL patients were standard R‐CHOP regimens.

**TABLE 1:** Clinical characteristics of 69 cases of diffuse large B‐cell lymphoma (DLBCL) in the Jiangsu Province Hospital (JSPH) cohort.

Characteristics	Total (*N*, %)
**Gender**	
Female	45 (65.2%)
Male	24 (34.8%)
**COO**	
GCB	26 (37.7%)
Non‐GCB	43 (62.3%)
**Age**	
≤60 years	45 (65.2%)
>60 years	24 (34.8%)
**Ann Arbor stage**	
I–II	37 (53.6%)
III–IV	32 (46.4%)
**Serum LDH levels**	
Normal	43 (62.3%)
Elevated	26 (37.7%)
**Extranodal locations**	
0–1	48 (69.6%)
>1	21 (30.4%)
**Performance status (ECOG PS)**	
0–1	58 (84.1%)
>1	11 (15.9%)
**IPI risk group**	
Low risk (0–1)	37 (53.6%)
Intermediated risk (2–3)	18 (26.1%)
High risk (4–5)	14 (20.3%)
**1st line response**	
CR/CMR	47 (68.1%)
PR	2 (2.9%)
SD	1 (1.5%)
PD	18 (26.1%)
NA	1 (1.5%)
**POD24**	
Good outcome	23 (52.3%)
Poor outcome	21 (47.7%)
**Risk group**	
High‐risk group	25 (36.2%)
Low‐risk group	44 (63.8%)

The analysis also encompassed two additional published cohorts of DLBCL that provided accessible clinical data, DNA mutation profiles, and gene expression data^7^, ^8^ (Table S1). All patients received the standard R‐CHOP regimen. Clinical data were gathered from the primary sources, and details regarding LymphGen subtypes were accessible within the GSE181063. The LymphGen subtype of GSE117556 was determined by employing the LymphGen implement (https://llmpp.nih.gov/lymphgen/index.php). The gene expressions of GSE117556 and GSE181063 were mixed into a single matrix file by utilizing the R package “sva”.

To curate a comprehensive list of PCD genes, we sourced genes linked to 19 distinct PCD patterns from esteemed scientific databases and literature. This compilation included Gene Set Enrichment Analysis (GSEA) gene sets, Kyoto Encyclopedia of Genes and Genome (KEGG) pathways, scholarly reviews, and a manual curation process. Post the removal of any redundant genes, our final dataset comprised 2118 unique PCD‐associated genes that were subsequently subjected to further analysis.^9^, ^10^, ^11^

### 2.2 Liquid biopsy data acquisition

The concentration of circulating tumour DNA (ctDNA) in each sample, expressed in human genomic equivalents per millilitre (hGE/mL), was determined by applying the subsequent formula: multiply the mean value of variant allele frequency (VAF) by the cell‐free DNA concentration (pg/mL plasma) and then divide by the haploid genomic equivalent weight of 3.3 pg. The results were expressed as base‐10 logarithms (log hGE/mL). Post‐infusion samples were qualitatively and quantitatively assessed for ctDNA at the end of treatment (EOT) and reported as negative or positive. In cases of a positive result, the concentration of ctDNA was quantified.

We focused on mutations identified in baseline plasma as potential disease biomarkers and investigated their presence in subsequent plasma samples after infusion. MRD status was determined: MRD positive indicated the re‐detection of any baseline or new mutation, whereas MRD negative signified the complete clearance of all baseline mutations.

### 2.3 Signature generated from machine learning‐based integrative approaches

To ascertain a reliable consensus on genes linked to pediatric cardiomyopathy (PCD) with precision and consistency, a comprehensive integration of 10 machine‐learning algorithms and 70 algorithmic combinations was employed. Within the training dataset, we developed 70 distinct machine learning‐based models, each trained using leave‐one‐out cross‐validation (LOOCV). Among these models, the combination of coxBoost and GBM was identified as the most suitable model, as it demonstrated the highest area under the curve (AUC) score in the validation dataset.

### 2.4 Tumour microenvironment analysis and drug sensitivity prediction

Expression profiles of key genes and the levels of various immune cell infiltrations were acquired and processed using a suite of computational algorithms. Furthermore, we employed the ‘oncoPredict’ R package to calculate the half‐maximal inhibitory concentration (IC50) for a range of chemotherapeutic agents, thereby identifying which drugs might be most effective for the high‐risk cohort.

### 2.5 Statistical analysis

Discrepancies among various risk groups were assessed using Unpaired Students' *t*‐tests, one‐way analysis of variance, Chi‐square analyses, or Fisher's exact tests, depending on the context. A *p*‐value of less than .05 was considered to indicate statistical significance. The data analyses were conducted utilizing R software (version 4.3.1), SPSS software (version 20.0) and GraphPad Prism (version 10.0).

The detailed methods of clinical data, genetic data, liquid biopsy data acquisition, a signature generated from machine learning‐based integrative approaches, tumour microenvironment analysis and drug sensitivity prediction, functional enrichment analysis, nomogram building and assessment, unsupervised clustering of model genes and statistical analysis were shown in Supporting Information methods.

## 3 RESULTS

### 3.1 Preliminary screening of PCD‐related regulators in less‐defined subtype DLBCL

**FIGURE 1:**
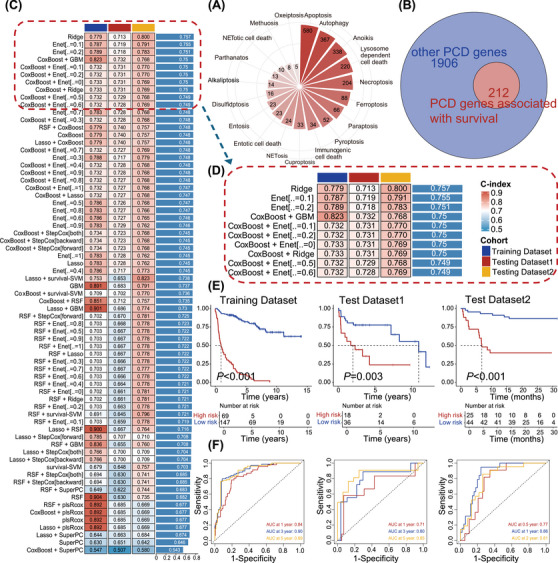
A consensus Programmed Cell Death Index (PCDI) was developed and validated via a multiple machine learning algorithms‐based integrative procedure. (A) Collection of key regulatory genes containing 19 PCD patterns. (B) A number of PCD genes associated with survival are displayed using a Venn plot. (C) A total of 70 prediction models were calculated using a 10‐fold cross‐validation framework to determine the C‐index of each model. (D) The top 10 prediction models ranked by the C‐index of test datasets. (E) Kaplan‐Meier analysis of progression‐free survival for less‐defined subtype patients with low‐ and high‐PCDI scores in the combined public database and Jiangsu Province Hospital (JSPH) cohort. (F) Receiver operator characteristic (ROC) analysis of 1‐, 3‐ and 5‐ PFS in the combined public database and JSPH cohort. PCD, programmed cell death; RSF, random survival forest; Enet, elastic network; plsRcox, partial least squares regression forex; SuperPC, supervised principal components; GBM, generalized boosted regression modelling; survival‐SVM, survival support vector machine.

The reanalysis involved integrating data from two bulk RNA sequencing cohorts, identified as GSE117556 (*N* = 116) and GSE181063 (*N* = 154), along with the JSPH cohort (*N* = 69). We curated a set of 2118 regulatory genes encompassing 19 PCD patterns sourced from literature (Figure 1A and Table S2). Patients from two public databases were divided in an 8:2 ratio based on PFS endpoint status, resulting in a training dataset of 216 patients and an internal test dataset of 54 patients. The JSPH cohort served as the external test dataset. Subsequently, we conducted univariate Cox regression analysis, identifying 212 regulatory genes associated with PFS in the training set (Figure 1B).

### 3.2 Integrative construction of a consensus signature

We utilized a suite of 10 distinct machine‐learning algorithms to develop the PCDI. In the training set, we constructed 70 machine learning models, employing LOOCV for training each one. The concordance index (C‐index) was utilized to assess the performance of the models. It was employed as a criterion for selecting the most effective model, focusing on the one that exhibited the highest mean C‐index across all validation datasets (Figure 1C). The coxBoost + GBM was selected among the top five performing model combinations for gene model selection (C‐index = 0.75, Figure 1D). Ultimately, our algorithm identified eight key PCDI signature genes: FLT3 (FMS‐like tyrosine kinase 3), SORL1 (sortilin‐related receptor 1), CD8A (cluster of differentiation 8A), COL13A1 (collagen type XIII alpha 1), BCL2L1 (BCL2‐like protein 1), MPG (methylpurine glycosylase), DYRK2 (dual‐specificity tyrosine‐(Y)‐phosphorylation regulated kinase 2), and CAMK2B (calcium/calmodulin‐dependent protein kinase II beta). Importantly, DLBCL patients from public databases with elevated expression of these signature genes exhibited prolonged survival (Figure S1A–H). Similar results were demonstrated in the JSPH cohort (Figure S1I–P).

Our analysis of patients diagnosed with less‐defined subtype DLBCL in the GSE101063, GSE117556, and JSPH cohort revealed that high PCDI scores were significantly associated with poorer prognosis, as depicted in Figure 1E (cutoff = 0.916, training dataset: *p *< .001, test dataset1: *p *= .003 and test dataset2: *p *< .001). Patients with high PCDI scores were classified as the high‐risk group, while the rest of the patients were designated as the low‐risk group. In the training cohort, the AUC scores were 0.84, 0.90, and 0.89 for each of the three consecutive years, respectively. The AUC scores, as indicated by the internal test dataset, were 0.71, 0.80 and 0.85, respectively. Furthermore, the external test dataset, known as the JSPH cohort, presented AUC scores of 0.77, 0.86 and 0.81 (Figure 1F). Furthermore, we performed univariate Cox analysis on various clinical features in public databases and the JSPH cohort. The results revealed survival differences among patients with different risk groups within subgroups defined by clinical features (Figure S2A,B). These findings underscore the prognostic significance of PCDI.

### 3.3 Characteristics annotation of PCDI signature genes

**FIGURE 2:**
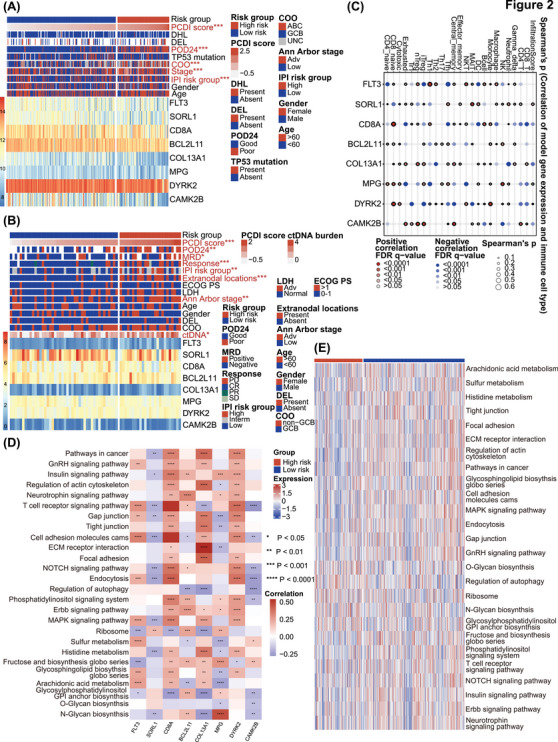
Annotation of characteristics for the PCDI signature genes. (A, B) Heatmaps of the PCDI prognostic model and clinical variables of public databases and the Jiangsu Province Hospital (JSPH) cohort. (C) Bubble plot presenting the correlation between the expression levels of eight PCDI signature genes and 24 immune cell types of infiltrates in less‐defined subtype diffuse large B‐cell lymphoma (DLBCL). (D) Heatmap showing the correlation between pathways and eight PCDI signature genes. (E) Heatmap displaying the enrichment scores of important pathways. PCDI, programmed cell death index; DHL, double hit lymphoma; DEL, double expression lymphoma; POD24, progression of disease within 2 years; COO, cell of origin; IPI, international prognostic index; MRD, minimal residual disease; ECOG PS, Eastern Cooperative Oncology Group performance status; LDH, lactate dehydrogenase; ctDNA, circulating tumour DNA.

Heatmaps of the PCDI‐related prognostic model and clinical characteristics of public databases and the JSPH cohort are depicted in Figures 2A and 2B. In public databases, discernible disparities were identified between low‐risk and high‐risk groups with respect to PCDI scores, progression of disease within 2 years (POD24), cell of origin (COO), Ann Arbor stage, and IPI risk group (*p *< .001 for all above), as determined by Chi‐square tests. The JSPH cohort showed similar results. Additionally, liquid biopsy analysis indicated higher pre‐treatment ctDNA concentrations in high‐risk patients (*p *< .05). At the end of therapy response evaluation, ctDNA was more frequently detected in the high‐risk group (*p* < .05). We also compared PCDI scores across different subgroups based on multiple clinical variables in both the combined public database and the JSPH cohort. In the combined public database, the advanced Ann Arbor stage and POD24 were associated with higher PCDI scores (Figure S3A–I). Similarly, in the JSPH cohort, non‐GCB subtype, advanced Ann Arbor stage, extranodal involvement > 1, IPI high‐risk group, PD/stable disease (SD) at the end of the first‐line therapy, MRD positivity, and POD24 correlated with higher PCDI scores (Figure S3J–T). We also analyzed the association between distinct risk groups and established markers of DLBCL. We observed a higher prevalence of whole blood EBV‐DNA positivity in the high‐risk group, which correlates with a poorer prognosis. However, we regret to report that no significant statistical differences were found in the expression of MYC, BCL2 and BCL6 between high‐risk and low‐risk groups in either the integrated cohort or the JSPH cohort (Table S3). Figure 2C provides an overview of the relationship between the expression of eight PCDI signature genes and the 24 distinct types of immune cells, encompassing 18 kinds of T cells along with other types of immune cells infiltrated in less‐defined subtype DLBCL. Additionally, we found that patients with high risk are more prone to exhibit higher central nervous system (CNS)‐IPI scores (*p *< .001), CNS involvement, and involvement of the adrenal glands, kidneys, and breasts, which are also associated with CNS involvement (Figure S4A,B). As illustrated in Figures 2D and 2E, several pathways significantly correlated with these 8 genes, encompassing position and regulation of PCD patterns, lipid metabolism, and various signalling pathways related to immunological regulation and LME.

### 3.4 The dynamic analysis of liquid biopsy between different PCDI groups

**FIGURE 3:**
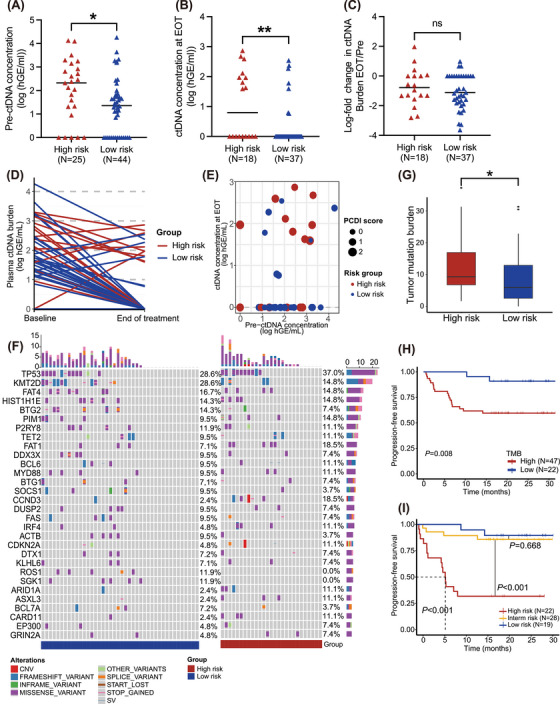
Dynamic analysis of liquid biopsy between different Programmed Cell Death Index (PCDI) groups. (A) Distribution of pretreatment plasma ctDNA concentration between low‐ and high‐risk groups. (B) Distribution of plasma ctDNA concentration at EOT between low‐ and high‐risk groups. (C) Distribution of log‐fold change in plasma ctDNA concentration between low‐ and high‐risk group. (D) Dynamic change in plasma ctDNA concentration from baseline to EOT depicted by a line plot. (E) Correlation between pretreatment plasma ctDNA concentration and ctDNA concentration at EOT. (F) Distribution of gene alterations in plasma samples. Each column represents a patient. (G) Boxplot of tumor mutation burden (TMB) between low‐ and high‐risk groups. (H) Kaplan‐Meier analysis of PFS for less‐defined subtype diffuse large B‐cell lymphoma (DLBCL) patients with low‐ and high‐TMB. (I) Kaplan‐Meier analysis of PFS for less‐defined subtype DLBCL patients with different risk groups combining TMB and PCDI groups. ctDNA, circulating tumour DNA; EOT, end of treatment; TMB, tumour mutation burden; PFS, progression‐free survival; CNV, copy number variation; SV, structural variation.

ctDNA is considered a novel prognostic and predictive biomarker with significant prognostic value for PFS and OS.^12^ Our results revealed that high‐risk patients typically exhibited higher ctDNA concentrations at pre‐treatment, and post‐treatment (Figures 3A and 3B). However, no significant difference was observed in the log‐fold change of ctDNA burden (Figure 3C). Through dynamic analysis, we detected that almost high‐risk patients tend to exhibit not only MRD positivity but also an elevated change of ctDNA burden (Figures 3D and 3E).

Tumour mutation analysis was performed on tissue and plasma samples diagnosed with less‐defined subtype DLBCL. Using the Fisher test, CCND3 mutations, associated with worse prognosis, were more prevalent in the plasma of the high‐risk group^13^ (Figure 3F and Table S4). Conversely, a higher frequency of BCL2 mutations was observed in the tissue samples of the low‐risk group, while NSD2 mutations which are associated with resistance of Imbruvica, were more frequently observed in the high‐risk group^14^, ^15^ (Figure S5 and Table S5). Additionally, in the combined public database, SGK1 mutations were more frequently observed in patients with low PCDI scores (Table S6).

We then compared TMB between individuals with high and low PCDI scores. To explore the link between TMB and PCDI, our findings revealed that the TMB was considerably elevated in the high‐risk subgroup, as depicted in Figure 3G. Notably, patients exhibiting reduced TMB demonstrated improved survival rates in contrast to those with elevated TMB levels, with a marked statistical disparity (*p *= .008, Figure 3H). To unravel the combined predictive value of TMB and PCDI for survival, we stratified patients according to these criteria and performed a survival analysis. Each factor's presence was allocated one point: a score of 2 was indicative of high risk, a score of 1 was of intermediate risk, and a score of 0 of low risk. Patients with low risk exhibited the most favourable prognosis, whereas those classified as high risk had the least favorable (low‐risk vs. intermediate‐risk, *p* = .668; intermediate‐risk vs. high‐risk, *p *< .001, Figure 3I).

### 3.5 The underlying biological mechanisms of PCDI groups

**FIGURE 4:**
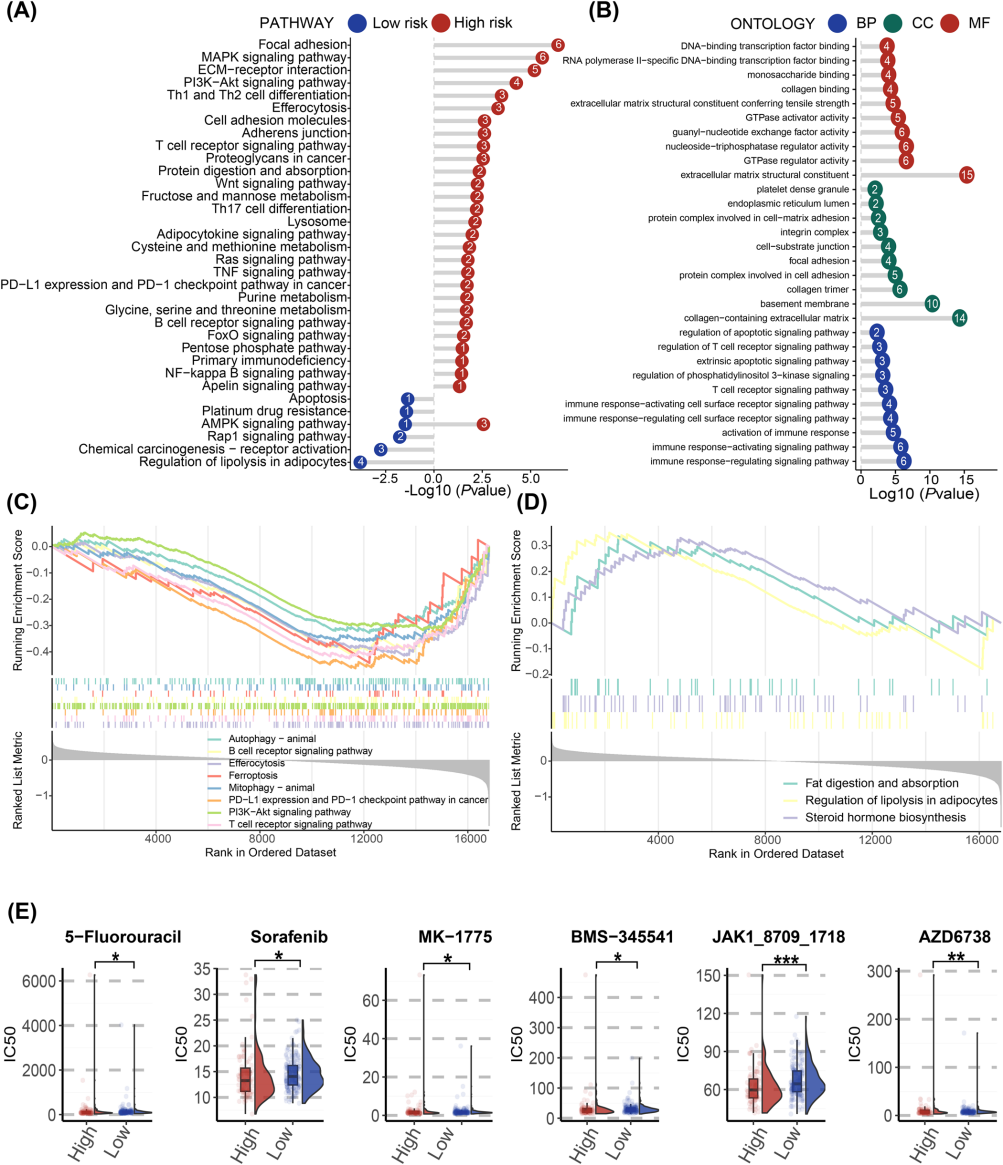
The underlying biological mechanisms of Programmed Cell Death Index (PCDI) groups. (A) Results of Kyoto Encyclopedia of Genes and Genomes (KEGG) analysis between low‐ and high‐risk patients. (B) Results of Gene Ontology (GO) analysis between low‐ and high‐risk patients. (C, D) Several important pathways are shown by Gene Set Enrichment Analysis (GSEA) between low‐ and high‐risk groups. (E) Distribution of IC50 for potential chemotherapeutic drugs between low‐ and high‐risk groups. BP, biological process; CC, cell component; MF, molecular function; KEGG, Kyoto Encyclopedia of Genes and Genomes; GO, Gene Ontology; GSEA, Gene Set Enrichment Analysis; IC50, half‐maximal inhibitory concentration.

To deepen our comprehension of the biological mechanisms associated with PCDI groups, we conducted a pathway enrichment analysis. The results of KEGG are shown in Figure 4A. In terms of cellular processes, PCDI were predominantly enriched in focal adhesion, lysosome, efferocytosis and adherens junction. For environmental information processing, PCDI were most highly concentrated in the PI3K‐Akt and NF‐kappa B signalling pathway. Additionally, PD‐L1 expression and PD‐1 checkpoint pathway were also enriched in the high‐risk group. The gene ontology (GO) results of patients with high risk are presented in Figure 4B.

Furthermore, we utilized GSEA to uncover potential pathways correlated with PCDI. As illustrated in Figure 4C and Figure 4E, the high‐risk patients showed significant enrichment in pathways similar to those identified by KEGG. To validate these results, we applied similar methods to patients in the JSPH cohort. Six of the eight regulatory genes have lower expression in individuals with high PCDI scores (Figure S6A). The results of KEGG and GSEA also exhibited disorder of several significant pathways, including Th1, Th2 and Th17 cell differentiation, JAK‐STAT, NF‐kappa B and PD‐L1 expression and PD‐1 checkpoint pathway in cancer (Figure S6C–E).

Based on the GDSC database, patients classified as high risk were found to be more responsive to a variety of specific anti‐neoplastic medications, such as 5‐Fluorouracil, sorafenib, MK‐1775, BMS‐345541, JAK1 inhibitor and AZD6738 (Figure 4E). Among the anti‐cancer drugs mentioned above, JAK1‐inhibitor showed a comparatively lower IC50 in patients in the high‐risk group compared to those in the low‐risk group.

### 3.6 Potential biological mechanisms in tumour immune process related to the PCDI signature

**FIGURE 5:**
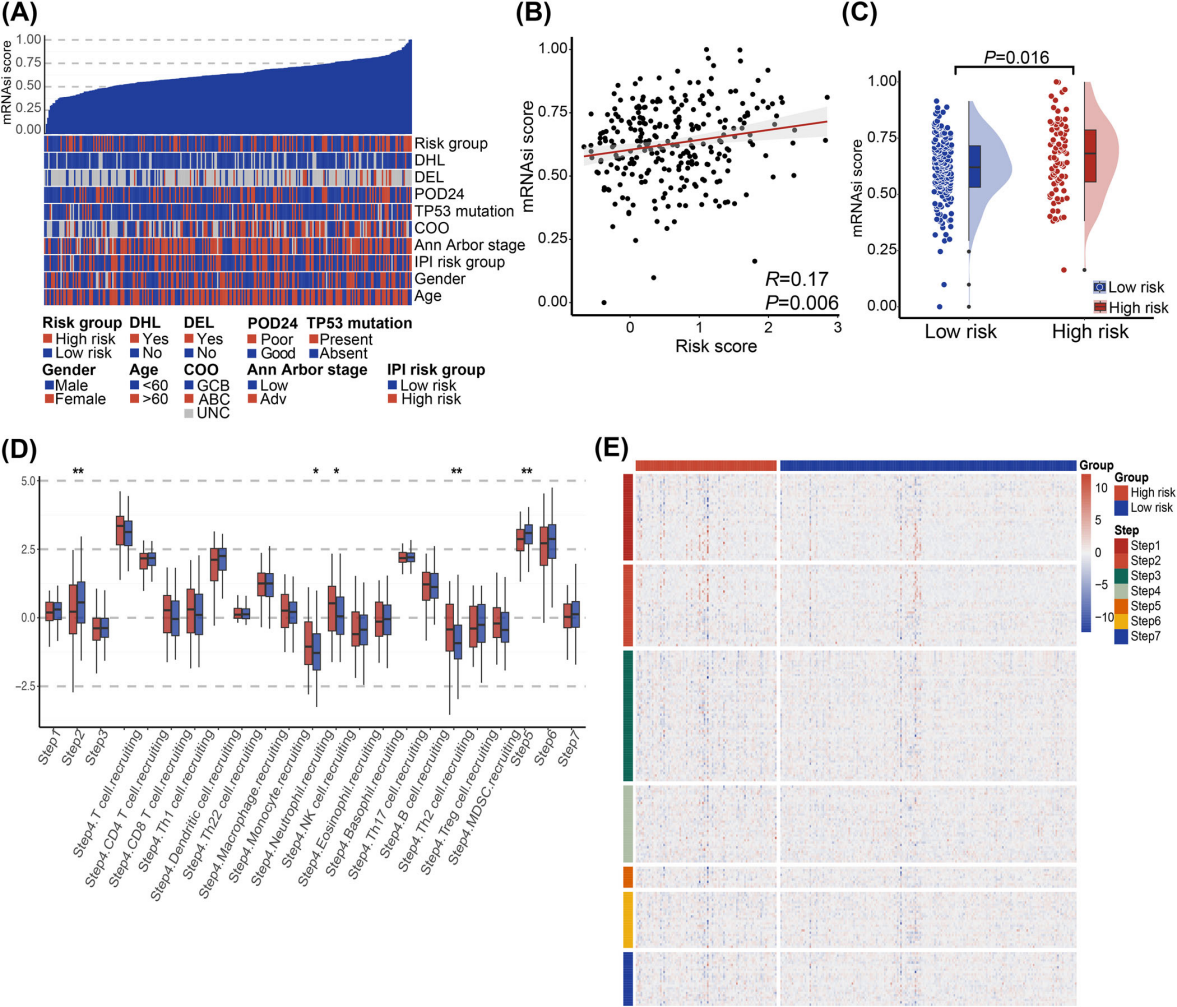
Potential biological mechanisms in tumour immune processes related to the PCDI signature. (A) Heatmap of mRNAsi scores and clinical features of public databases. (B) Correlation between mRNAsi scores and PCDI scores. (C) Distribution of mRNAsi scores between patients with low and high risk. (D) Box plot portraying the dissimilarities in the cancer immunity cycle between two PCDI groups. (E) Heatmap illustrating the expression levels of 178 step‐specific signature genes associated with anti‐cancer immunity across all samples in the seven‐step Cancer‐Immunity Cycle. DHL double hit lymphoma; DEL, double expression lymphoma; PCDI, programmed cell death index; POD24, progression of disease within 2 years; IPI, international prognostic index.

Cancer stem cells (CSCs) constitute a minor subset of cells within a lymphoma that share characteristics with normal stem cells, notably their capacity to differentiate into the various cell types present within a specific tumour sample. In our quest to explore the gene expression profiles and epigenetic signatures of CSCs, we determined mRNAsi scores in samples retrieved from publicly accessible databases. mDNAsi scores were not calculated due to the lack of DNA methylation data. A heatmap of mRNAsi scores and clinical characters is displayed in Figure 5A. No significant correlation between risk scores and mRNAsi scores was observed (Figure 5B). However, higher mRNAsi scores can be observed in patients with high risk. (*p *= .016, Figure 5C).

The development of an antitumor immune response involves a series of steps, succinctly encapsulated within the framework of the “cancer immunity cycle^16^”. In our quest to explore the biological mechanisms underlying the PCDI signature, we assessed the trajectory of cancer immunity. Notably, within the cohort identified as high‐risk, disruptions were observed in three distinct stages of the tumour immune cycle: phase 2 (cancer antigen presentation), phase 4 (tumour immunized infiltrating cells recruitment) and phase 5 (immune tissues influx) (Figure 5D). Furthermore, the levels of expression for 178 genes linked to protective mechanisms against cancer, spanning the seven phases of the cancer‐immunity process, are illustrated in Figure 5E.

### 3.7 Immune characteristics between distinct PCDI groups

**FIGURE 6:**
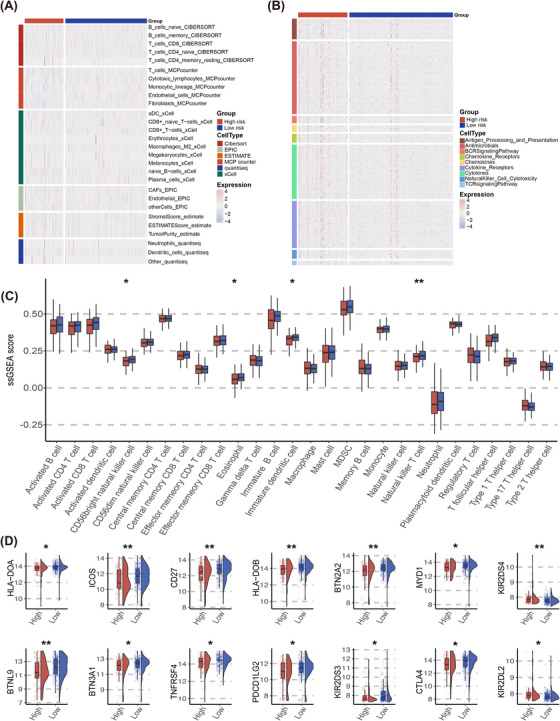
Immune characteristics between distinct PCDI groups. (A) Immune infiltrating cells estimated using multiple algorithms between low‐ and high‐risk groups. (B) Heatmap illustrating the expression levels of 2499 immune‐related signature genes. (C) Distribution of ssGSEA scores between low‐ and high‐risk groups. (D) Boxplots comparing the expression levels of several immune checkpoints between low‐ and high‐risk groups. ssGSEA, single‐sample gene set enrichment analysis; TIMER, tumour immune estimation resource; CIBERSORT, cell‐type identification by estimating relative subpopulations of RNA transcripts; MCPcounter, microenvironment cell populations‐counter; EPIC, estimation of proportion of immune and cancer cells.

To assess the characteristics of the tumour microenvironment between different PCDI groups, we examined the presence of immune cell infiltration utilizing seven distinct algorithms (Figure 6A). Low PCDI scores were associated with higher infiltration of CD4+ T cells, CD8+ T cells and M1 macrophage. Patients exhibiting high PCDI scores had notably reduced Stromal and ESTIMATE scores. Similar results were observed in patients from the JSPH cohort grouped by PCDI scores (Table S7). Furthermore, we employed the ssGSEA enrichment score to investigate the correlation between the PCDI signature and various immune cell subsets as well as their functional activities. Our results showed that among CD56 bright natural killer cell, eosinophil, natural killer T cell and immature dendritic cell, ssGSEA scores of patients with high PCDI scores were significantly different from those with low PCDI scores for these immune‐related cell functions (Figure 6C). In the JSPH cohort, central memory CD4 T cell was more commonly observed in the low‐risk group (Figure S7). Additionally, PCDI characteristic scores were negatively correlated with immune checkpoints, including HLA‐DOA, HLA‐DOB, ICOS, CD27, CTLA4, PD‐L2 and so on (Figure 6D).

### 3.8 Development and evaluation of the nomogram prognostic model

**FIGURE 7:**
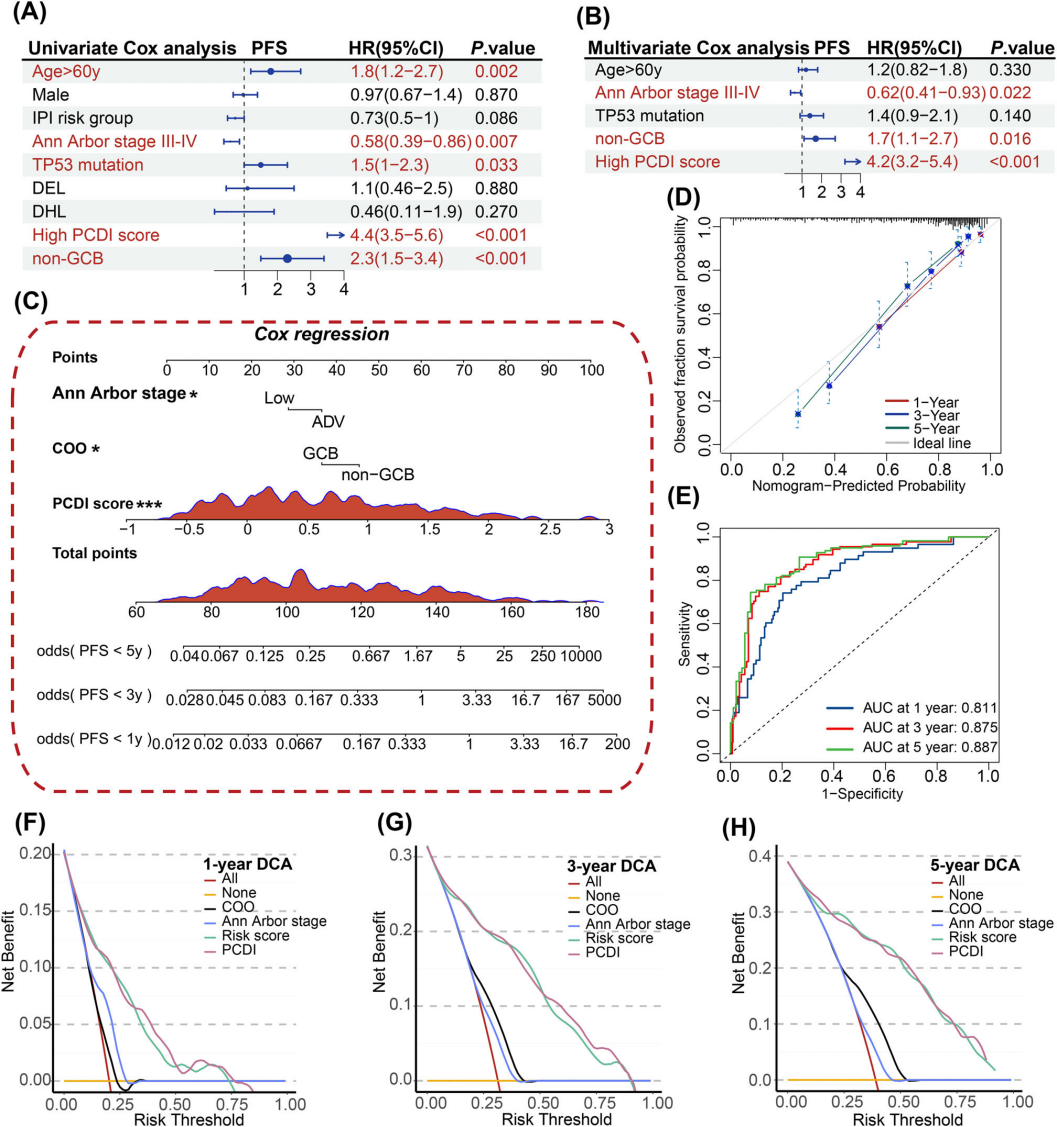
Development and evaluation of the nomogram survival model. (A, B) Univariate and multivariate analysis of clinical characteristics and PCDI in public databases. (C) A nomogram model was established to predict the prognosis of patients from public databases. (D) Calibration plots showing the probability of 1‐, 3‐ and 5‐year PFS in the combined public database. (E) Receiver operating characteristic (ROC) analysis of the nomogram model in the combined public database. (F–H) Decision curve analysis (DCA) of the nomogram predicting 1‐, 3‐ and 5‐year PFS. PCDI, programmed cell death index; DEL, double expression lymphoma; DHL, double hit lymphoma; COO, cell of origin; PFS, progression‐free survival; ROC, receiver operating characteristic; AUC, area under curve; DCA, decision curve analysis.

**FIGURE 8:**
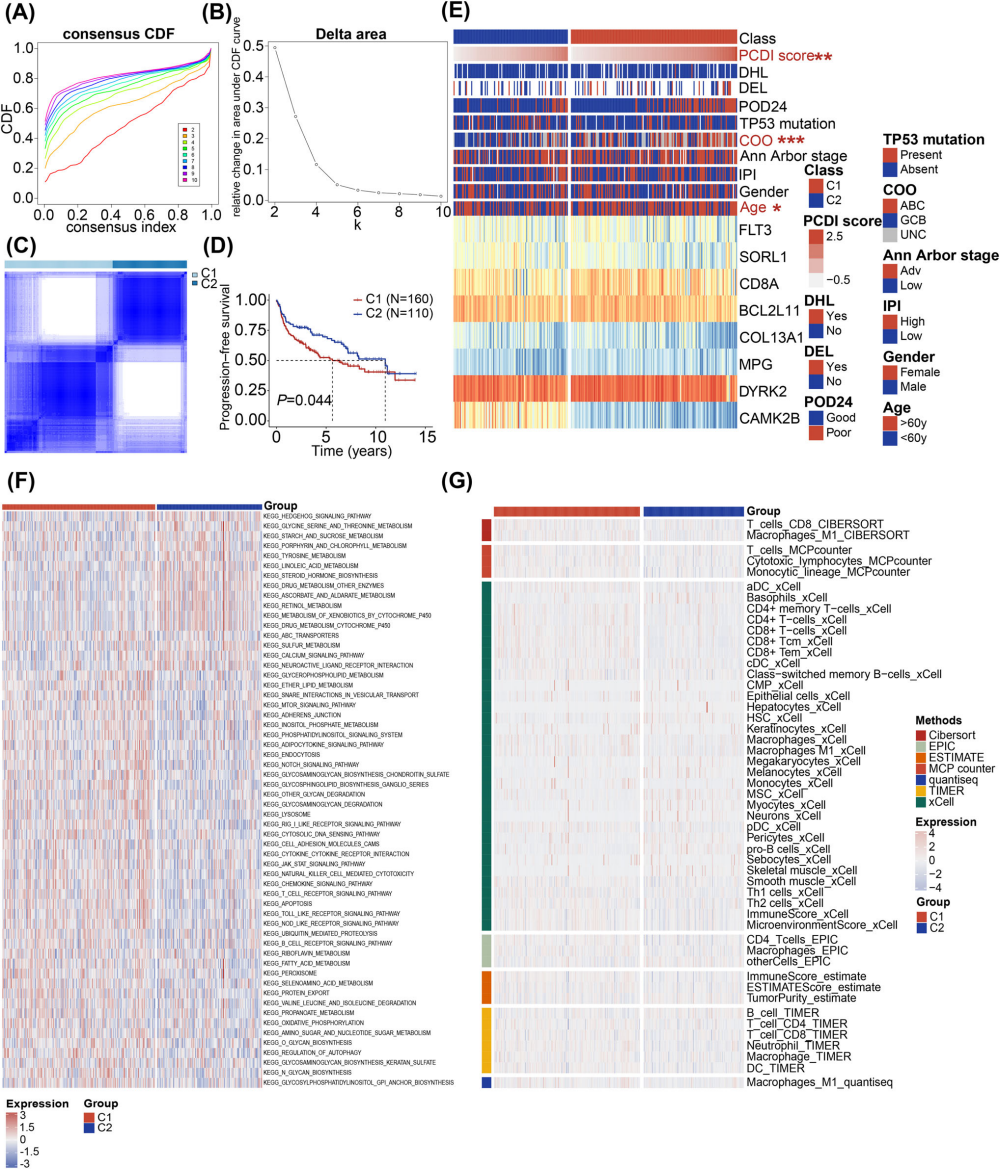
Consensus clustering to identify two clusters of patients. (A, B) Consensus clustering CDF for k = 2–10. (C) Consensus clustering with k = 2. (D) Kaplan‐Meier survival curves of PFS among the two subgroups. (E) Heatmap showing the clinical features and expression of 8 signature genes in less‐defined subtype diffuse large B‐cell lymphoma (DLBCL) patients between the two subgroups. (F) Heatmap displaying enrichment scores of important pathways between the two subgroups. (G) Immune infiltrating cells estimated using multiple algorithms between the two subgroups. CDF, cumulative distribution function; PCDI, programmed cell death index; DHL, double hit lymphoma; DEL, double expression lymphoma; POD24, progression of disease within 2 years; COO, cell of origin; IPI, international prognostic index; ssGSEA, single‐sample gene set enrichment analysis; TIMER, tumour immune estimation resource; CIBERSORT, cell‐type identification by estimating relative subpopulations of RNA transcripts; MCPcounter, microenvironment cell populations‐counter; EPIC, estimation of proportion of immune and cancer cells.

To evaluate the prognostic impact of PCDI independently, separate univariate and multivariate Cox regression analyses were conducted. The results indicated that PCDI emerged as a substantial risk factor in the univariate Cox regression analysis (hazard ratio [HR] = 4.4, 95% confidence interval [95%CI]: 3.5–5.6, *p *< .001, Figure 7A). When considering multivariate analysis, the PCDI continued to show its independent prognostic significance in patients with less‐defined subtype DLBCL, even after considering other potential influencing factors (HR = 1.7, 95%CI: 1.1–2.7, *p *< .001, Figure 7B). Utilizing multivariable Cox and stepwise regression analyses, a prognostic nomogram was established within public databases to estimate the 1‐, 3‐ and 5‐year PFS for patients with less‐defined subtype DLBCL (Figure 7C). Given the results of our study, we have developed an online tool for risk assessment (https://xulymphoma.shinyapps.io/PCDI_pred/). This user‐friendly platform is designed to efficiently evaluate risks and serves as a basis for the creation of a practical algorithm suitable for clinical use. The accuracy of the nomogram model's predictive capabilities for 1‐, 3‐ and 5‐year survival rates was confirmed through calibration and ROC curve analysis (Figure 7D,E). Furthermore, decision curve analysis (DCA) indicated that our nomogram model's predictive performance surpassed that of other models in the study (Figure 7F–H). Similar results were observed in the JSPH cohort (Figure 8A–E).

### 3.9 Consensus clustering to identify two clusters of patients

To investigate the role of PCDI signature genes in the progression of less‐defined subtype DLBCL, we undertook an exhaustive clustering analysis focusing on the activity values associated with the eight signature indicators. K = 2 was determined to be optimal stable clustering (Figure 8A,B). This analysis divided the samples from public databases into two subgroups: C1 (*N* = 160) and C2 (*N* = 110) (Figure 8C). Notably, there were plenty of variations in PFS between the two subgroups (*p *= .044, Figure 8D). C1 was predominantly composed of patients with high PCDI scores, while C2 was mainly composed of those with low PCDI scores (*p *= .006). Furthermore, the heatmap depicting PCDI consensus clusters highlighted significant disparities between clusters C1 and C2 sections in terms of risk score, COO and age (Figure 8E). Moreover, the pathway‐based Gene Set Variation Analysis (GSVA) findings revealed that the C1 subtype exhibited increased activation of pathways related to tumours and immunity, including the T cell receptor, B cell receptor, and JAK‐STAT signalling pathway, along with endocytosis and apoptosis. This suggests a strong correlation between the PCDI signature and these canonical tumour‐associated pathways (Figure 8F). Additionally, the difference in immune cell infiltration is depicted in Figure 8G.

## 4 DISCUSSION

In our research, we discovered 212 PCD genes correlated with survival outcomes and constructed a consensus prognostic PCDI utilizing various machine learning algorithms with a dataset comprising 270 patients diagnosed with less‐defined subtype DLBCL from public databases and 69 from the JSPH cohort. Incorporating both the PCDI and clinical features, we constructed a prognostic nomogram model leveraging data from public databases, demonstrating robust and reliable predictive performance. Notably, our study identified a substantial correlation between the PCDI and the LME, along with drug sensitivity in a less‐defined subtype of DLBCL. These findings underscore the PCDI's potential utility in clinical practice for tailoring therapeutic strategies to individual patient needs.

It is widely recognized that diverse patterns of PCD are intricately linked to the development and metastasis of lymphomas. In this study, we have crafted a signature including eight genes related to PCD (FLT3, SORL1, CD8A, BCL2L11, COL13A1, MPG, DYRK2 and CAMK2B) utilizing data from 339 patients diagnosed with less‐defined subtype DLBCL across multiple public datasets and the JSPH cohort. Subsequently, we embarked on an extensive bioinformatics analysis to explore the genetic terrain and clinical significance of these model genes within the context of less‐defined subtype DLBCL.

FLT3 is a receptor tyrosine kinase primarily expressed in hematopoietic cells and involved in cell proliferation, differentiation, and survival.^17^ In 2019, Douglas et al. found that the reduction of SORL1 expression promotes tumour growth in a tumour transplant model.^18^ CD8A encodes a protein known as the CD8 alpha chain, which is a component of the CD8 molecule. An abundance of CD8+ T cell infiltration is frequently linked to improved prognosis.^19^ BCL2L11 is crucial in the modulation of apoptosis, maintaining T‐ and B‐cell homeostasis. BCL2L11 encodes the Bim protein, which promotes apoptosis by interacting with and neutralizing anti‐apoptotic BCL‐2 family members.^20^ COL13A1 is involved in the signalling pathways between the extracellular matrix and cells, influencing cell growth, differentiation, and apoptosis.^21^ The protein encoded by the MPG gene is essential in the base excision repair (BER) pathway. The MPG enzyme recognizes and excises abnormal or damaged bases from the DNA strand, thereby initiating the repair process.^22^, ^23^ During the past 10 years, DYRK2 has been identified as a tumour suppressor in a range of malignancies, initiating significant antitumor and proapoptotic responses. Decreased expression of DYRK2 expression has been linked to a worse prognosis for patients.^24^, ^25^

Liquid biopsies that detect ctDNA have the ability to revolutionize the personalized management of lymphoma. To evaluate the correlation of the PCDI and ctDNA concentration, 69 newly diagnosed patients diagnosed with less‐defined subtype DLBCL underwent ctDNA analysis at diagnosis and at the end of treatment. We observed that the high‐risk group exhibited a higher ctDNA burden at diagnosis and at the end of treatment than those classified as a low‐risk group, which is consistent with poor outcomes. TMB refers to the count of somatic mutations per megabase (Mb) within a tumour's genome, serving as an indicator of genomic instability. Tumours characterized by a high TMB are more prone to stimulate the generation of neoantigens, which can render them susceptible to immune cell recognition and attack.^26^, ^27^, ^28^ The high‐risk group consistently exhibited higher TMB, which correlated with poor outcomes.

In order to evaluate the clinical significance of the PCDI in less‐defined subtype DLBCL, we formulated a nomogram model that integrates the PCDI with pertinent clinical variables. The subsequent validation of this model substantiated its effectiveness, highlighting its practical utility in a clinical setting. Notably, advanced Ann Arbor stage, non‐GCB subtype and high PCDI score were determined to independently prognostic factors. In contrast to the machine learning‐based model developed by Jia, our approach focused on the heterogeneity in the prognosis of patients diagnosed with less‐defined subtypes, where current research fails to distinguish high‐risk patients effectively. By integrating various cell death pathways and employing diverse machine learning techniques, we have successfully identified patients with the truly high‐risk group with less‐defined subtype DLBCL. Furthermore, we propose a tailored treatment strategy for these patients, which is a novel contribution not previously reported in the literature.^3^, ^29^

Patients exhibiting lower PCDI scores demonstrated a notably higher survival rate when contrasted with those who had higher PCDI scores. The prognostic nomogram model, which incorporates the PCDI, has proven to possess robust predictive capabilities for estimating the 1‐, 3‐ and 5‐year PFS in patients diagnosed with less‐defined subtype DLBCL. However, due to the heterogeneity of less‐defined subtype DLBCL, further comprehensive investigations are essential to elucidate the role of the PCDI model within a larger cohort. The necessary studies for further exploration should encompass mechanistic research, clinical trials, as well as Supporting Information clinical assessments.

As we know, Lymphoma cells have the capacity to avoid immune detection and counteract the impact of therapeutic agents, which facilitates their survival and advancement.^30^, ^31^, ^32^, ^33^ Our research revealed significant differences in the biological mechanism and LME based on PCDI levels. Patients with high PCDI scores exhibited enrichment of multiple pathways correlated to lymphoma cell proliferation, including PI3K‐Akt, JAK‐STAT and NF‐kappa B signalling pathways. Additionally, we observed the activation of PD‐L1 expression and PD‐1 checkpoint pathway. This raises the question of whether immune checkpoint inhibitor (ICI) therapy or inhibitors of specific signalling pathways are effective in improving the prognosis of high‐risk patients. This research shed light on the correlation between PCDI scores and drug responsiveness in patients with less‐defined subtype DLBCL. Based on the GDSC database, JAK1 inhibitors demonstrate the highest significance. The JACKPOT8 PART B clinical trial demonstrated that Golidocitinib can significantly improve the prognosis of peripheral T‐cell lymphoma (PTCL) patients.^34^ However, the clinical value of Golidocitinib in B‐cell lymphoma remains controversial. Additionally, PD‐1 inhibitors are considered potentially effective for high‐risk patients with less‐defined subtype DLBCL due to the activation of PD‐L1 expression and PD‐1 checkpoint pathway, the activation of JAK‐STAT signalling pathway, which can lead to PD‐1 overexpression, and the low mRNA expression of the PD‐L2 gene, the ligand for PD‐1. Our results provide a potential theoretical basis for the application of these drugs in patients diagnosed with less‐defined subtype DLBCL.

In the analysis of LME, we found that lymphomas with elevated PCDI scores were associated with a diminished presence of immune cells that combat tumours, such as M1 macrophage, CD4+ T cells, and CD8+ T cells. In contrast, there was an increase in immunosuppressive cells like fibroblasts and M2 macrophages in lymphomas with high PCDI scores. The observed inverse relationship between PCDI and effector immune cells suggests that lymphomas with elevated PCDI scores may present with a phenotype that is more immunosuppressed.^35^, ^36^ Harnessing the presence of immune infiltrates strategically could enhance the effectiveness of immunotherapy, presenting an opportunity to reduce the negative impact of a pro‐tumoral environment. Additionally, a detailed examination of immune checkpoint expression yielded more understanding of the weakened immune response observed in patients with elevated PCDI scores. A decrease in inhibitory receptors such as KIR2DS4, KIR2DS3, KIR2DL2, CTLA‐4, and PD‐L2 was detected, which may stem from the exhaustion of NK and T cells due to ongoing immune suppression. These observations collectively imply a worsening of NK and T cell dysfunction.^37^

Our research indeed sheds light on the clinical relevance of the PCDI signature. However, it is crucial to recognize the study's inherent limitations. Firstly, although the analysis included a retrospective database and the JSPH cohort, the sample size of the JSPH cohort should be expanded to confirm the accuracy of the model. Given the complex nature of less‐defined subtype DLBCL and the heterogeneity in their treatment responses, it is essential to undertake further extensive mechanistic and clinical research to elucidate the function of PCD genes in less‐defined subtype DLBCL.

In conclusion, we have successfully developed a PCDI signature using data from public databases and confirmed its effectiveness through validation within the JSPH cohort, showcasing its enhanced predictive capabilities. Nonetheless, it is essential that further research be undertaken to overcome the limitations previously discussed in order to bolster the robustness and practical use of our results.

## AUTHOR CONTRIBUTIONS

**Conception and design**: Rui Gao; Jin‐Hua Liang and Wei Xu. **Collection of study materials or patients’ data**: All authors. **Assembly of data and data analysis**: Jin‐Hua Liang; Wei Hua; Jie Liu and Jun‐Heng Liang. **Manuscript writing and editing**: Jin‐Hua Liang; Wei Hua; Jun‐Heng Liang and Wei Xu. **Final approval of manuscript**: All authors. **Accountable for all aspects of the work**: All authors.

## CONFLICT OF INTEREST STATEMENT

The authors declare no conflict of interest.

## ETHICS STATEMENT

This study was conducted in accordance with the Declaration of Helsinki, and the study protocol was approved by the Ethics Committee of the Institutional Review Broad of Jiangsu Province Hospital (No. 2023‐SR‐190) and informed consent was retrieved from subjects involved in this study. We have also obtained informed consent for the publication of the involved images with anonymization.

## Supporting information



Supporting Information

Supporting Information

Supporting Information

## Data Availability

We agree to share publication‐related data. Sequencing data that the reported findings of the study are based on are being uploaded to a public database.

## References

[1] Wright GW , Huang DaW , Phelan JD , et al. A probabilistic classification tool for genetic subtypes of diffuse large B cell lymphoma with therapeutic implications. Cancer Cell. 2020;37(4):551‐568.e14. 32289277 10.1016/j.ccell.2020.03.015 PMC8459709

[2] Sehn LH , Gascoyne RD . Diffuse large B‐cell lymphoma: optimizing outcome in the context of clinical and biologic heterogeneity. Blood. 2015;125(1):22‐32. 25499448 10.1182/blood-2014-05-577189

[3] Zhang Mu‐C , Tian S , Fu Di , et al. Genetic subtype‐guided immunochemotherapy in diffuse large B cell lymphoma: the randomized GUIDANCE‐01 trial. Cancer Cell. 2023;41(10):1705‐1716.e5. 37774697 10.1016/j.ccell.2023.09.004

[4] Peng Fu , Liao M , Qin R , et al. Regulated cell death (RCD) in cancer: key pathways and targeted therapies. Signal Transduct Target Ther. 2022;7(1):286. 35963853 10.1038/s41392-022-01110-y PMC9376115

[5] Zheng X , Jin X , Ye F , et al. Ferroptosis: a novel regulated cell death participating in cellular stress response, radiotherapy, and immunotherapy. Exp Hematol Oncol. 2023;12(1):65. 37501213 10.1186/s40164-023-00427-w PMC10375783

[6] Bedoui S , Herold MJ , Strasser A . Emerging connectivity of programmed cell death pathways and its physiological implications. Nat Rev Mol Cell Biol. 2020;21(11):678‐695. 32873928 10.1038/s41580-020-0270-8

[7] Painter D , Barrans S , Lacy S , et al. Cell‐of‐origin in diffuse large B‐cell lymphoma: findings from the UK's population‐based Haematological Malignancy Research Network. Br J Haematol. 2019;185(4):781‐784. 30408148 10.1111/bjh.15619

[8] Sha C , Barrans S , Cucco F , et al. Molecular high‐grade B‐Cell lymphoma: defining a poor‐risk group that requires different approaches to therapy. J Clin Oncol. 2019;37(3):202‐212. 30523719 10.1200/JCO.18.01314 PMC6338391

[9] Zou Y , Xie J , Zheng S , et al. Leveraging diverse cell‐death patterns to predict the prognosis and drug sensitivity of triple‐negative breast cancer patients after surgery. Int J Surg. 2022;107:106936. 36341760 10.1016/j.ijsu.2022.106936

[10] Zheng P , Zhou C , Ding Y , Duan S . Disulfidptosis: a new target for metabolic cancer therapy. J Exp Clin Cancer Res. 2023;42(1):103. 37101248 10.1186/s13046-023-02675-4 PMC10134647

[11] Wang X , Wang Z , Guo Z , Wang Z , Chen F , Wang Z . Exploring the role of different cell‐death‐related genes in sepsis diagnosis using a machine learning algorithm. Int J Mol Sci. 2023;24(19):14720. 37834169 10.3390/ijms241914720 PMC10572834

[12] Zhang Qu , Luo J , Wu S , et al. Prognostic and predictive impact of circulating tumor DNA in patients with advanced cancers treated with immune checkpoint blockade. Cancer Discov. 2020;10(12):1842‐1853. 32816849 10.1158/2159-8290.CD-20-0047 PMC8358981

[13] Hua W , Li Y , Yin H , et al. Analysis of CCND3 mutations in diffuse large B‐cell lymphoma. Ann Hematol. Published online: June 18, 2024. 10.1007/s00277-024-05844-3 38886191

[14] Narang S , Evensen NA , Saliba J , et al. NSD2 E1099K drives relapse in pediatric acute lymphoblastic leukemia by disrupting 3D chromatin organization. Genome Biol. 2023;24(1):64. 37016431 10.1186/s13059-023-02905-0 PMC10071675

[15] Jain P , Wang ML . Mantle cell lymphoma in 2022‐A comprehensive update on molecular pathogenesis, risk stratification, clinical approach, and current and novel treatments. Am J Hematol. 2022;97(5):638‐656. 35266562 10.1002/ajh.26523

[16] Chen DS , Mellman I . Oncology meets immunology: the cancer‐immunity cycle. Immunity. 2013;39(1):1‐10. 23890059 10.1016/j.immuni.2013.07.012

[17] Gilliland DG , Griffin JD . The roles of FLT3 in hematopoiesis and leukemia. Blood. 2002;100(5):1532‐1542. 12176867 10.1182/blood-2002-02-0492

[18] Michael IP , Saghafinia S , Hanahan D . A set of microRNAs coordinately controls tumorigenesis, invasion, and metastasis. Proc Natl Acad Sci U S A. 2019;116(48):24184‐24195. 31704767 10.1073/pnas.1913307116 PMC6883852

[19] Raskov H , Orhan A , Christensen JP , Gögenur I . Cytotoxic CD8(+) T cells in cancer and cancer immunotherapy. Br J Cancer. 2021;124(2):359‐367. 32929195 10.1038/s41416-020-01048-4 PMC7853123

[20] Kapoor I , Bodo J , Hill BT , Hsi ED , Almasan A . Targeting BCL‐2 in B‐cell malignancies and overcoming therapeutic resistance. Cell Death Dis. 2020;11(11):941. 33139702 10.1038/s41419-020-03144-y PMC7608616

[21] Zhang H , Fredericks T , Xiong G , et al. Membrane associated collagen XIII promotes cancer metastasis and enhances anoikis resistance. Breast Cancer Res. 2018;20(1):116. 30285809 10.1186/s13058-018-1030-y PMC6167877

[22] Chakravarti D , Ibeanu GC , Tano K , Mitra S . Cloning and expression in *Escherichia coli* of a human cDNA encoding the DNA repair protein N‐methylpurine‐DNA glycosylase. J Biol Chem. 1991;266(24):15710‐15715. 1874728

[23] Hedglin M , O'brien PJ . Human alkyladenine DNA glycosylase employs a processive search for DNA damage. Biochemistry. 2008;47(44):11434‐11445. 18839966 10.1021/bi801046y PMC2702167

[24] Van Den Boom J , Wolter M , Kuick R , et al. Characterization of gene expression profiles associated with glioma progression using oligonucleotide‐based microarray analysis and real‐time reverse transcription‐polymerase chain reaction. Am J Pathol. 2003;163(3):1033‐1043. 12937144 10.1016/S0002-9440(10)63463-3 PMC1868272

[25] Zhang Y , Xu J , Zhu X . A 63 signature genes prediction system is effective for glioblastoma prognosis. Int J Mol Med. 2018;41(4):2070‐2078. 29393370 10.3892/ijmm.2018.3422 PMC5810221

[26] Chen C , Liu S , Jiang X , et al. Tumor mutation burden estimated by a 69‐gene‐panel is associated with overall survival in patients with diffuse large B‐cell lymphoma. Exp Hematol Oncol. 2021;10(1):20. 33722306 10.1186/s40164-021-00215-4 PMC7962318

[27] Chalmers ZR , Connelly CF , Fabrizio D , et al. Analysis of 100,000 human cancer genomes reveals the landscape of tumor mutational burden. Genome Med. 2017;9(1):34. 28420421 10.1186/s13073-017-0424-2 PMC5395719

[28] Chen Y , Wang Y , Luo H , et al. The frequency and inter‐relationship of PD‐L1 expression and tumour mutational burden across multiple types of advanced solid tumours in China. Exp Hematol Oncol. 2020;9:17. 32775040 10.1186/s40164-020-00173-3 PMC7397649

[29] Jia Z , Zhang J , Li Z , Ai L . Identification of ferroptosis‐related genes associated with diffuse large B‐cell lymphoma via bioinformatics and machine learning approaches. Int J Biol Macromol. 2024;282(Pt 3):137117. 39488307 10.1016/j.ijbiomac.2024.137117

[30] Mohme M , Riethdorf S , Pantel K . Circulating and disseminated tumour cells—mechanisms of immune surveillance and escape. Nat Rev Clin Oncol. 2017;14(3):155‐167. 27644321 10.1038/nrclinonc.2016.144

[31] Dustin ML . The immunological synapse. Cancer Immunol Res. 2014;2(11):1023‐1033. 25367977 10.1158/2326-6066.CIR-14-0161 PMC4692051

[32] Garrido F , Cabrera T , Aptsiauri N . “Hard” and “soft” lesions underlying the HLA class I alterations in cancer cells: implications for immunotherapy. Int J Cancer. 2010;127(2):249‐256. 20178101 10.1002/ijc.25270

[33] Challa‐Malladi M , Lieu YK , Califano O , et al. Combined genetic inactivation of β2‐Microglobulin and CD58 reveals frequent escape from immune recognition in diffuse large B cell lymphoma. Cancer Cell. 2011;20(6):728‐740. 22137796 10.1016/j.ccr.2011.11.006 PMC3660995

[34] Song Y , Malpica L , Cai Q , et al. Golidocitinib, a selective JAK1 tyrosine‐kinase inhibitor, in patients with refractory or relapsed peripheral T‐cell lymphoma (JACKPOT8 Part B): a single‐arm, multinational, phase 2 study. Lancet Oncol. 2024;25(1):117‐125. 38092009 10.1016/S1470-2045(23)00589-2

[35] Chen DS , Mellman I . Elements of cancer immunity and the cancer‐immune set point. Nature. 2017;541(7637):321‐330. 28102259 10.1038/nature21349

[36] Zhang T , Liu H , Jiao L , et al. Genetic characteristics involving the PD‐1/PD‐L1/L2 and CD73/A2aR axes and the immunosuppressive microenvironment in DLBCL. J Immunother Cancer. 2022;10(4):e004114. 35365585 10.1136/jitc-2021-004114 PMC8977791

[37] Zhang Q , Bi J , Zheng X , et al. Blockade of the checkpoint receptor TIGIT prevents NK cell exhaustion and elicits potent anti‐tumor immunity. Nat Immunol. 2018;19(7):723‐732. 29915296 10.1038/s41590-018-0132-0

