# Supplementary material for: Revealing the heterogeneity of treatment resistance in less‐defined subtype diffuse large B cell lymphoma patients by integrating programmed cell death patterns and liquid biopsy
Source: Clin Transl Med. 2024 Dec 27;15(1):e70150. doi: 10.1002/ctm2.70150 (PMC11680560; doi:10.1002/ctm2.70150)
Supplement: Supplementary file 1 — Supporting Information [file CTM2-15-e70150-s001.pdf]

Figure S1

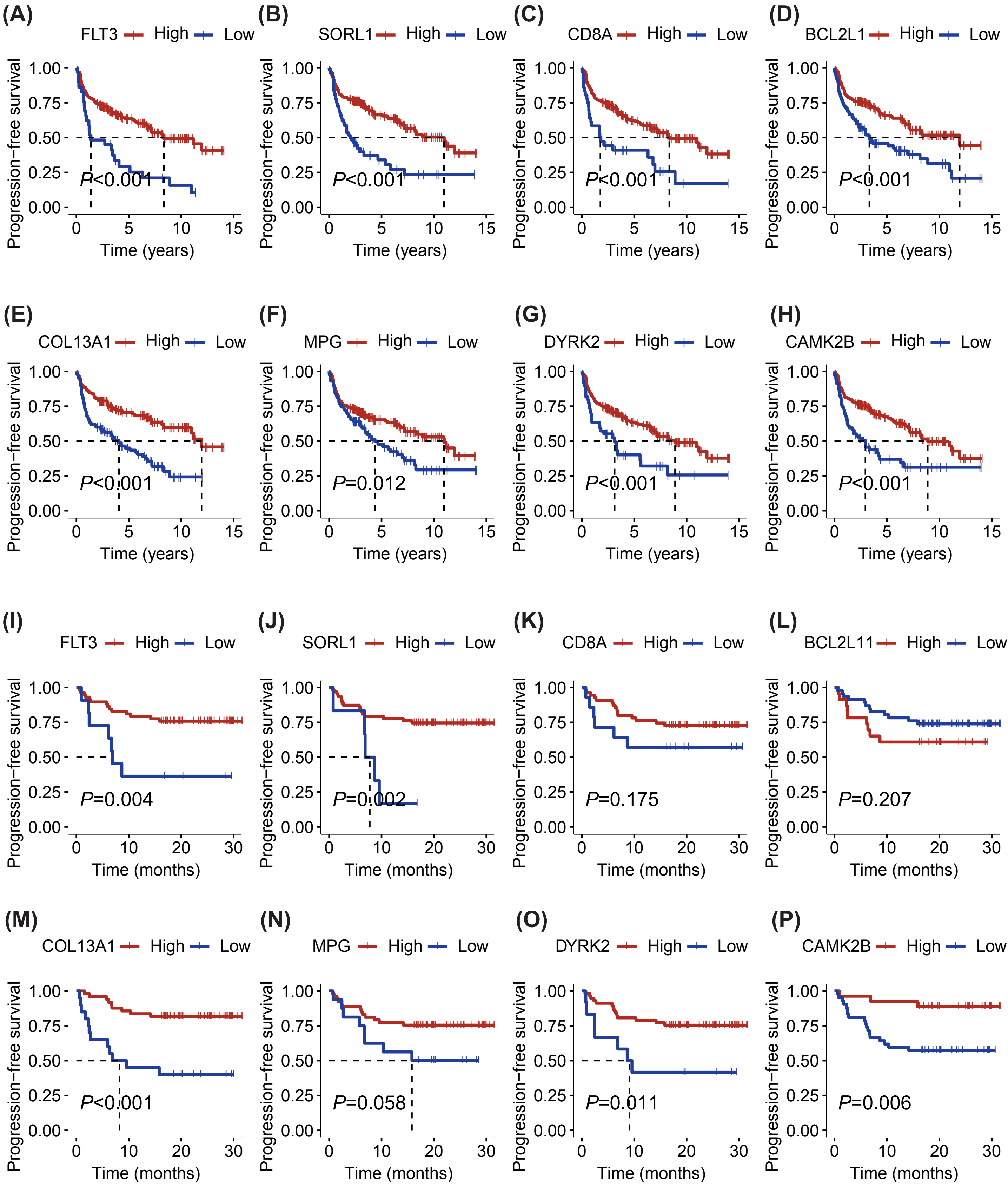

(A)
FLT3 High Low
P<0.001
(B)
SORL1 High Low
P<0.001
(C)
CD8A High Low
P<0.001
(D)
BCL2L1 High Low
P<0.001
(E)
COL13A1 High Low
P<0.001
(F)
MPG High Low
P=0.012
(G)
DYRK2 High Low
P<0.001
(H)
CAMK2B High Low
P<0.001
(I)
FLT3 High Low
P=0.004
(J)
SORL1 High Low
P=0.002
(K)
CD8A High Low
P=0.175
(L)
BCL2L11 High Low
P=0.207
(M)
COL13A1 High Low
P<0.001
(N)
MPG High Low
P=0.058
(O)
DYRK2 High Low
P=0.011
(P)
CAMK2B High Low
P=0.006
Progression−free survival
Time (years)
Time (months)

Figure S2

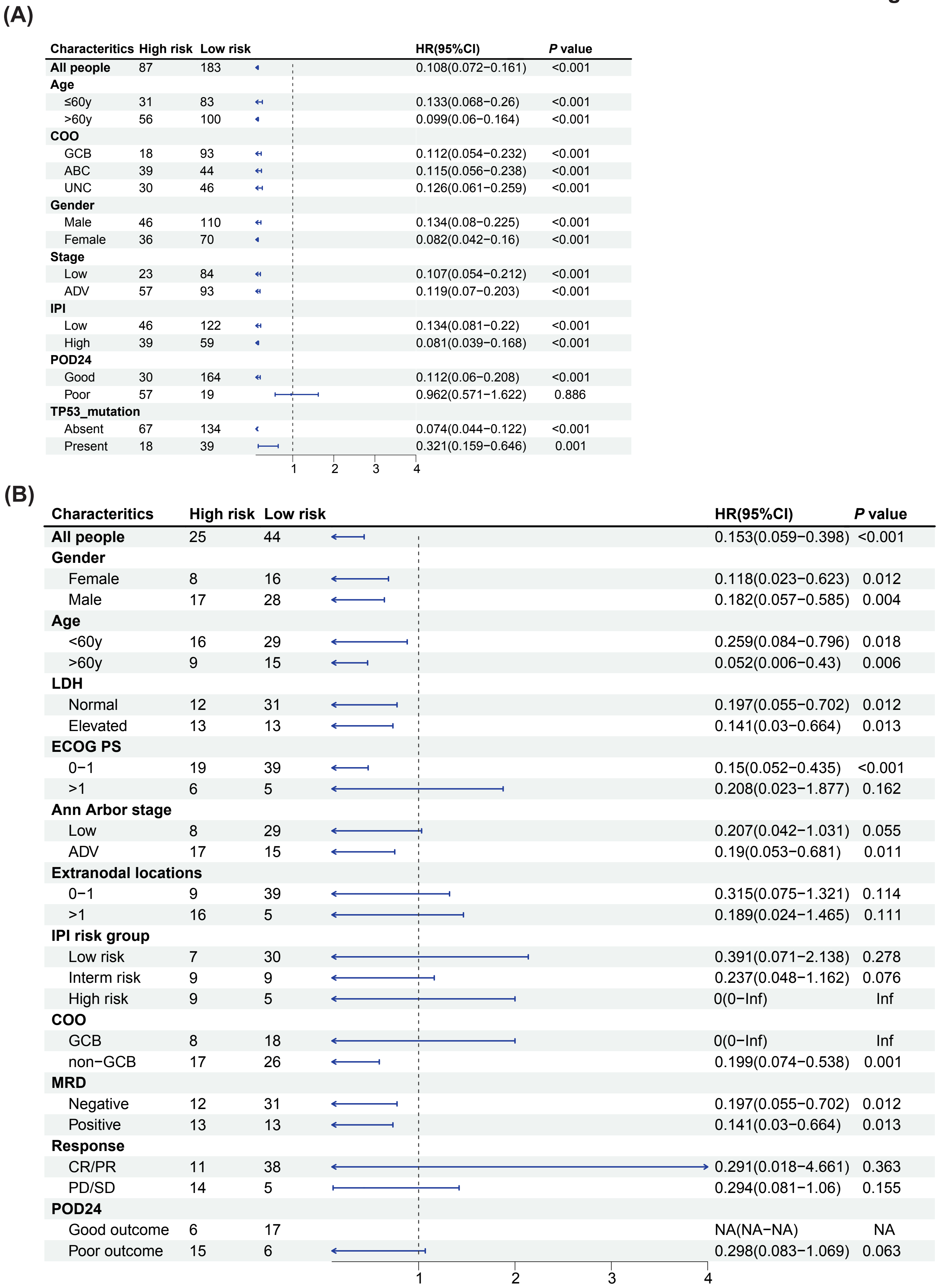
(A)
Characteritics High risk Low risk HR(95%CI) P value
All people 87 183 0.108(0.072−0.161) <0.001
Age
≤60y 31 83 0.133(0.068−0.26) <0.001
>60y 56 100 0.099(0.06−0.164) <0.001
COO
GCB 18 93 0.112(0.054−0.232) <0.001
ABC 39 44 0.115(0.056−0.238) <0.001
UNC 30 46 0.126(0.061−0.259) <0.001
Gender
Male 46 110 0.134(0.08−0.225) <0.001
Female 36 70 0.082(0.042−0.16) <0.001
Stage
Low 23 84 0.107(0.054−0.212) <0.001
ADV 57 93 0.119(0.07−0.203) <0.001
IPI
Low 46 122 0.134(0.081−0.22) <0.001
High 39 59 0.081(0.039−0.168) <0.001
POD24
Good 30 164 0.112(0.06−0.208) <0.001
Poor 57 19 0.962(0.571−1.622) 0.886
TP53_mutation
Absent 67 134 0.074(0.044−0.122) <0.001
Present 18 39 0.321(0.159−0.646) 0.001
1 2 3 4
(B)
Characteritics High risk Low risk HR(95%CI) P value
All people 25 44 0.153(0.059−0.398) <0.001
Gender
Female 8 16 0.118(0.023−0.623) 0.012
Male 17 28 0.182(0.057−0.585) 0.004
Age
<60y 16 29 0.259(0.084−0.796) 0.018
>60y 9 15 0.052(0.006−0.43) 0.006
LDH
Normal 12 31 0.197(0.055−0.702) 0.012
Elevated 13 13 0.141(0.03−0.664) 0.013
ECOG PS
0−1 19 39 0.15(0.052−0.435) <0.001
>1 6 5 0.208(0.023−1.877) 0.162
Ann Arbor stage
Low 8 29 0.207(0.042−1.031) 0.055
ADV 17 15 0.19(0.053−0.681) 0.011
Extranodal locations
0−1 9 39 0.315(0.075−1.321) 0.114
>1 16 5 0.189(0.024−1.465) 0.111
IPI risk group
Low risk 7 30 0.391(0.071−2.138) 0.278
Interm risk 9 9 0.237(0.048−1.162) 0.076
High risk 9 5 0(0−Inf) Inf
COO
GCB 8 18 0(0−Inf) Inf
non−GCB 17 26 0.199(0.074−0.538) 0.001
MRD
Negative 12 31 0.197(0.055−0.702) 0.012
Positive 13 13 0.141(0.03−0.664) 0.013
Response
CR/PR 11 38 0.291(0.018−4.661) 0.363
PD/SD 14 5 0.294(0.081−1.06) 0.155
POD24
Good outcome 6 17 NA(NA−NA) NA
Poor outcome 15 6 0.298(0.083−1.069) 0.063
1 2 3 4

Figure S3

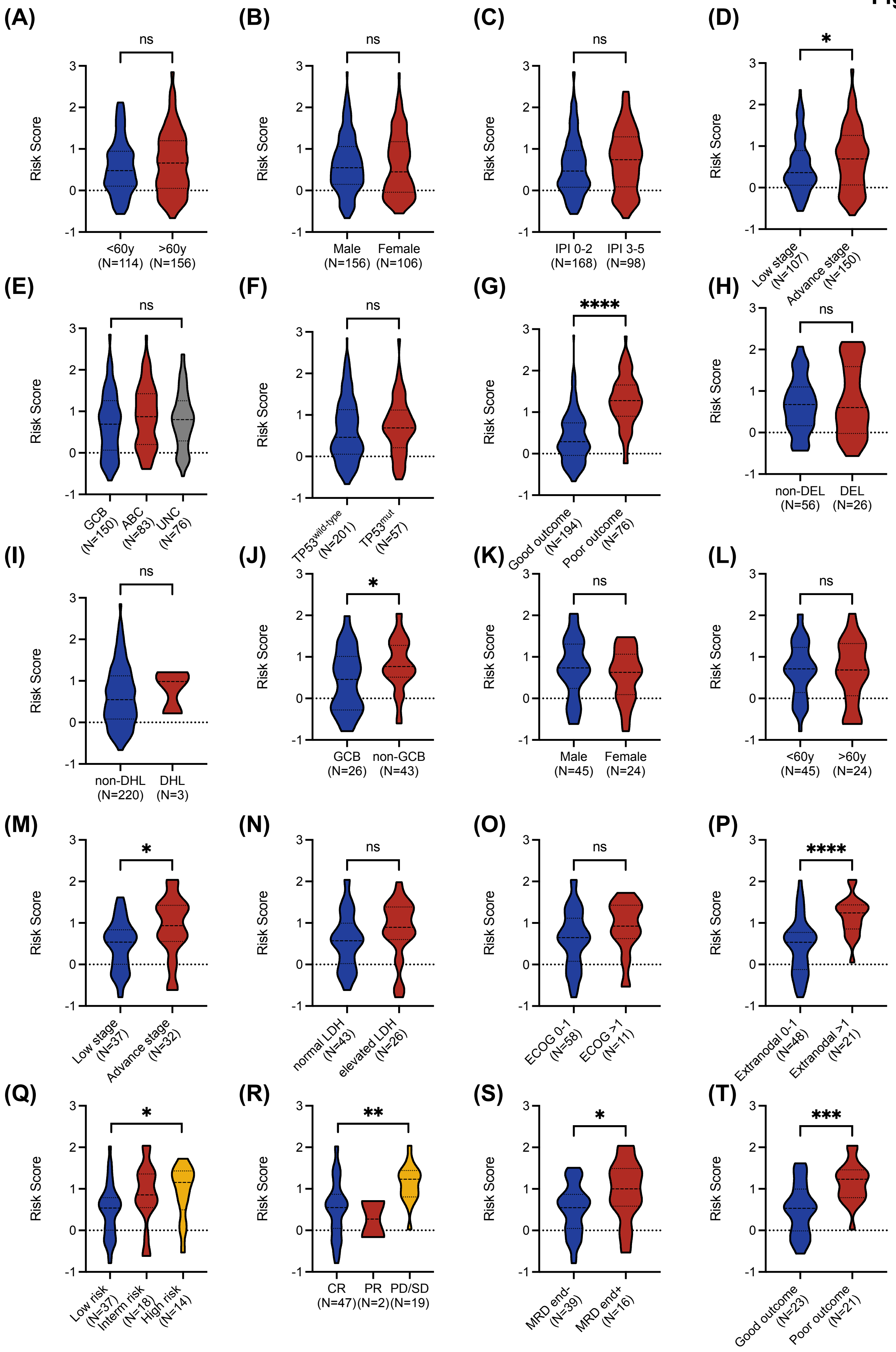

(A)
ns
Risk Score
<60y (N=114)
>60y (N=156)
(B)
ns
Male (N=156)
Female (N=106)
(C)
ns
IPI 0-2 (N=168)
IPI 3-5 (N=98)
(D)
*
Low stage (N=107)
Advance stage (N=150)
(E)
ns
GCB (N=150)
ABC (N=83)
UNC (N=76)
(F)
ns
TP53wild-type (N=201)
TP53mut (N=57)
(G)
****
Good outcome (N=194)
Poor outcome (N=76)
(H)
ns
non-DEL (N=56)
DEL (N=26)
(I)
ns
non-DHL (N=220)
DHL (N=3)
(J)
*
GCB (N=26)
non-GCB (N=43)
(K)
ns
Male (N=45)
Female (N=24)
(L)
ns
<60y (N=45)
>60y (N=24)
(M)
*
Low stage (N=37)
Advance stage (N=32)
(N)
ns
normal LDH (N=43)
elevated LDH (N=26)
(O)
ns
ECOG 0-1 (N=58)
ECOG >1 (N=11)
(P)
****
Extranodal 0-1 (N=48)
Extranodal >1 (N=21)
(Q)
*
Low risk (N=37)
Interm risk (N=18)
High risk (N=14)
(R)
**
CR (N=47)
PR (N=2)
PD/SD (N=19)
(S)
*
MRD end- (N=39)
MRD end+ (N=16)
(T)
***
Good outcome (N=23)
Poor outcome (N=21)

Figure S4

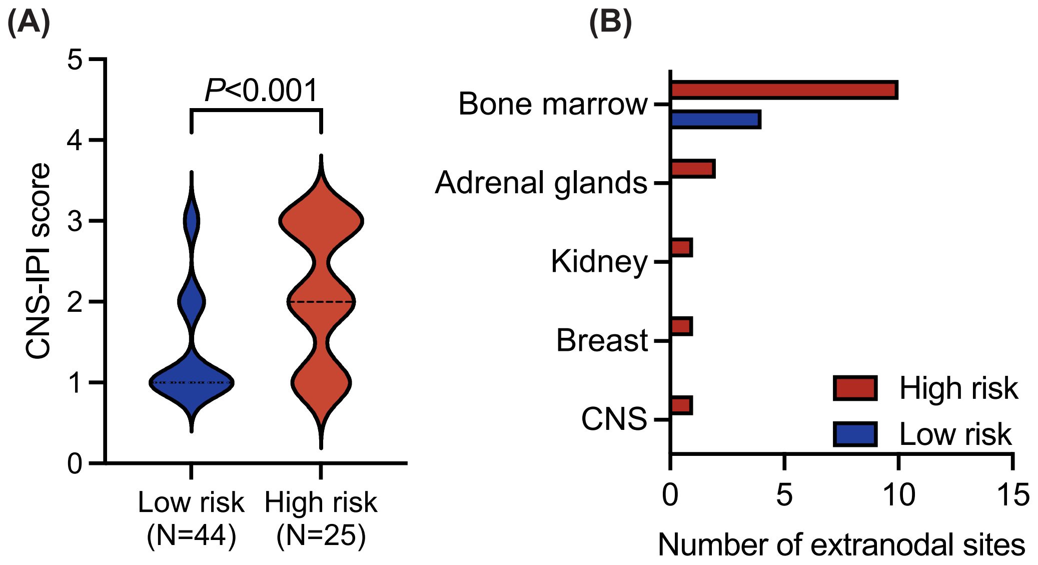
(A)
P<0.001
CNS-IPI score
0
1
2
3
4
5
Low risk (N=44)
High risk (N=25)
(B)
Bone marrow
Adrenal glands
Kidney
Breast
CNS
High risk
Low risk
0
5
10
15
Number of extranodal sites

Figure S5

(A)

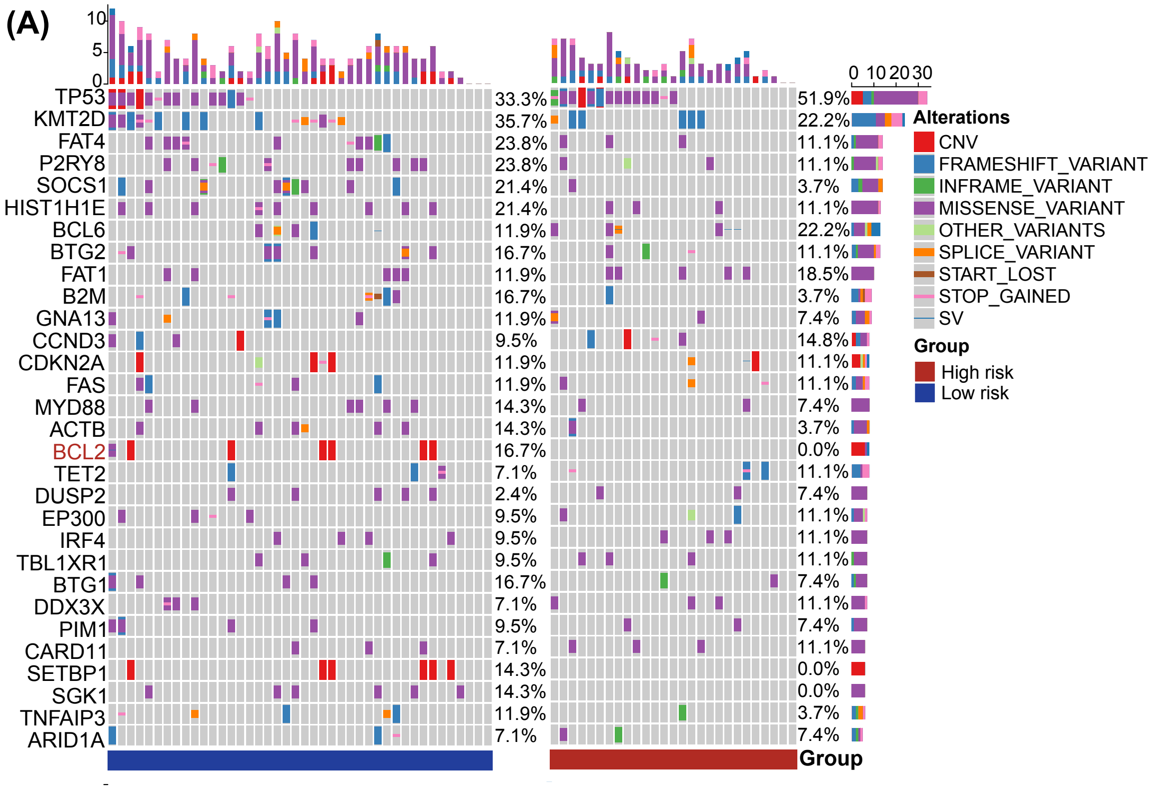
TP53
KMT2D
FAT4
P2RY8
SOCS1
HIST1H1E
BCL6
BTG2
FAT1
B2M
GNA13
CCND3
CDKN2A
FAS
MYD88
ACTB
BCL2
TET2
DUSP2
EP300
IRF4
TBL1XR1
BTG1
DDX3X
PIM1
CARD11
SETBP1
SGK1
TNFAIP3
ARID1A
33.3%
35.7%
23.8%
23.8%
21.4%
21.4%
11.9%
16.7%
11.9%
16.7%
11.9%
9.5%
11.9%
11.9%
14.3%
14.3%
16.7%
7.1%
2.4%
9.5%
9.5%
9.5%
16.7%
7.1%
9.5%
7.1%
14.3%
14.3%
11.9%
7.1%
51.9%
22.2%
11.1%
11.1%
3.7%
11.1%
22.2%
11.1%
18.5%
3.7%
7.4%
14.8%
11.1%
11.1%
7.4%
3.7%
0.0%
11.1%
7.4%
11.1%
11.1%
11.1%
7.4%
11.1%
7.4%
11.1%
0.0%
0.0%
3.7%
7.4%
Group
Alterations
CNV
FRAMESHIFT_VARIANT
INFRAME_VARIANT
MISSENSE_VARIANT
OTHER_VARIANTS
SPLICE_VARIANT
START_LOST
STOP_GAINED
SV
Group
High risk
Low risk

Figure S6

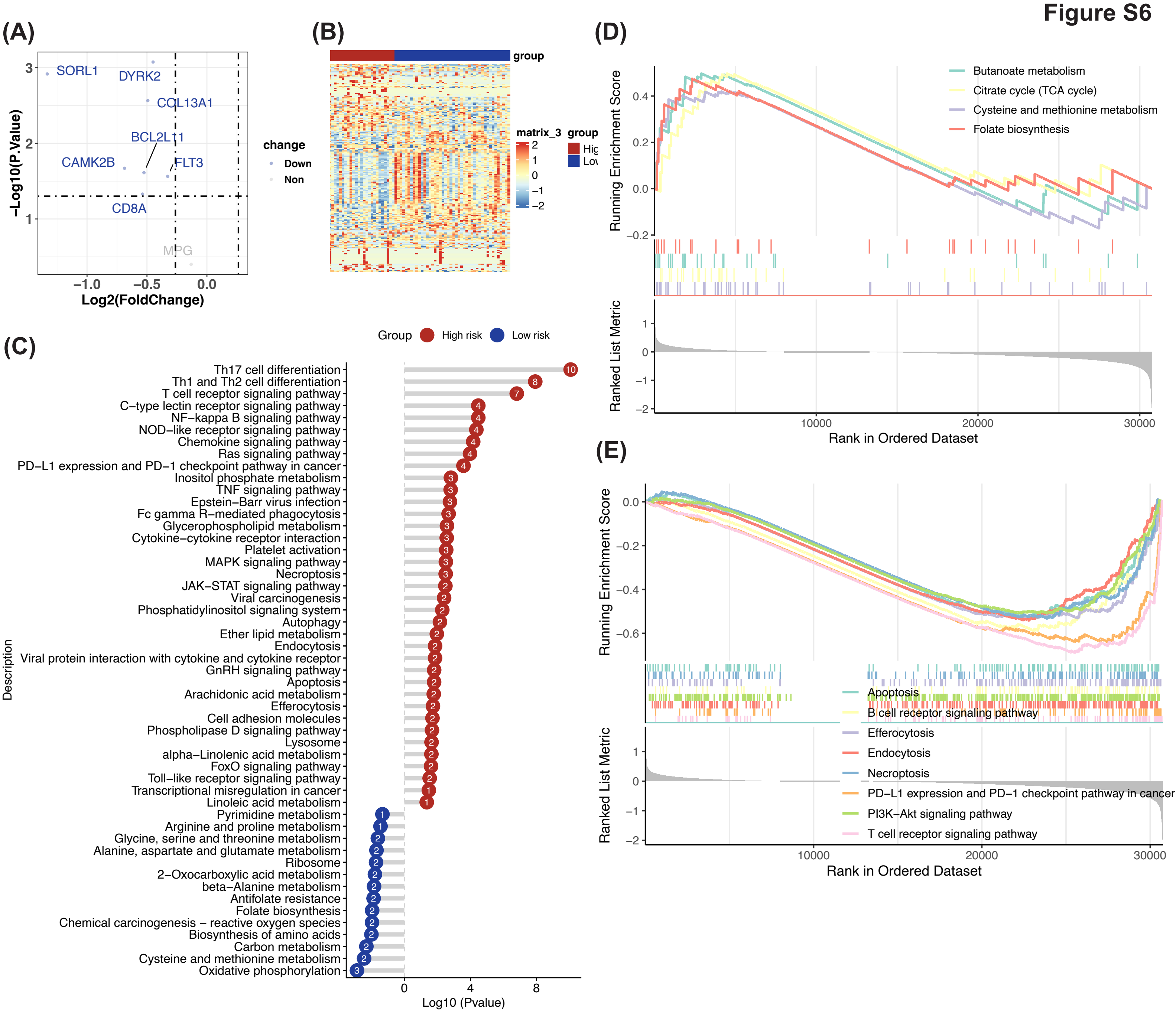

(A)
SORL1
DYRK2
CCL13A1
BCL2L11
CAMK2B
FLT3
CD8A
MPG
change
Down
Non
−Log10(P.Value)
Log2(FoldChange)
(B)
group
matrix_3
group
(C)
Group
High risk
Low risk
Th17 cell differentiation
Th1 and Th2 cell differentiation
T cell receptor signaling pathway
C−type lectin receptor signaling pathway
NF−kappa B signaling pathway
NOD−like receptor signaling pathway
Chemokine signaling pathway
Ras signaling pathway
PD−L1 expression and PD−1 checkpoint pathway in cancer
Inositol phosphate metabolism
TNF signaling pathway
Epstein−Barr virus infection
Fc gamma R−mediated phagocytosis
Glycerophospholipid metabolism
Cytokine−cytokine receptor interaction
Platelet activation
MAPK signaling pathway
Necroptosis
JAK−STAT signaling pathway
Viral carcinogenesis
Phosphatidylinositol signaling system
Autophagy
Ether lipid metabolism
Endocytosis
Viral protein interaction with cytokine and cytokine receptor
GnRH signaling pathway
Apoptosis
Arachidonic acid metabolism
Efferocytosis
Cell adhesion molecules
Phospholipase D signaling pathway
Lysosome
alpha−Linolenic acid metabolism
FoxO signaling pathway
Toll−like receptor signaling pathway
Transcriptional misregulation in cancer
Linoleic acid metabolism
Pyrimidine metabolism
Arginine and proline metabolism
Glycine, serine and threonine metabolism
Alanine, aspartate and glutamate metabolism
Ribosome
2−Oxocarboxylic acid metabolism
beta−Alanine metabolism
Antifolate resistance
Folate biosynthesis
Chemical carcinogenesis − reactive oxygen species
Biosynthesis of amino acids
Carbon metabolism
Cysteine and methionine metabolism
Oxidative phosphorylation
Description
Log10 (Pvalue)
(D)
Butanoate metabolism
Citrate cycle (TCA cycle)
Cysteine and methionine metabolism
Folate biosynthesis
Running Enrichment Score
Ranked List Metric
Rank in Ordered Dataset
(E)
Apoptosis
B cell receptor signaling pathway
Efferocytosis
Endocytosis
Necroptosis
PD−L1 expression and PD−1 checkpoint pathway in cancer
PI3K−Akt signaling pathway
T cell receptor signaling pathway
Running Enrichment Score
Ranked List Metric
Rank in Ordered Dataset

Figure S7

(A)

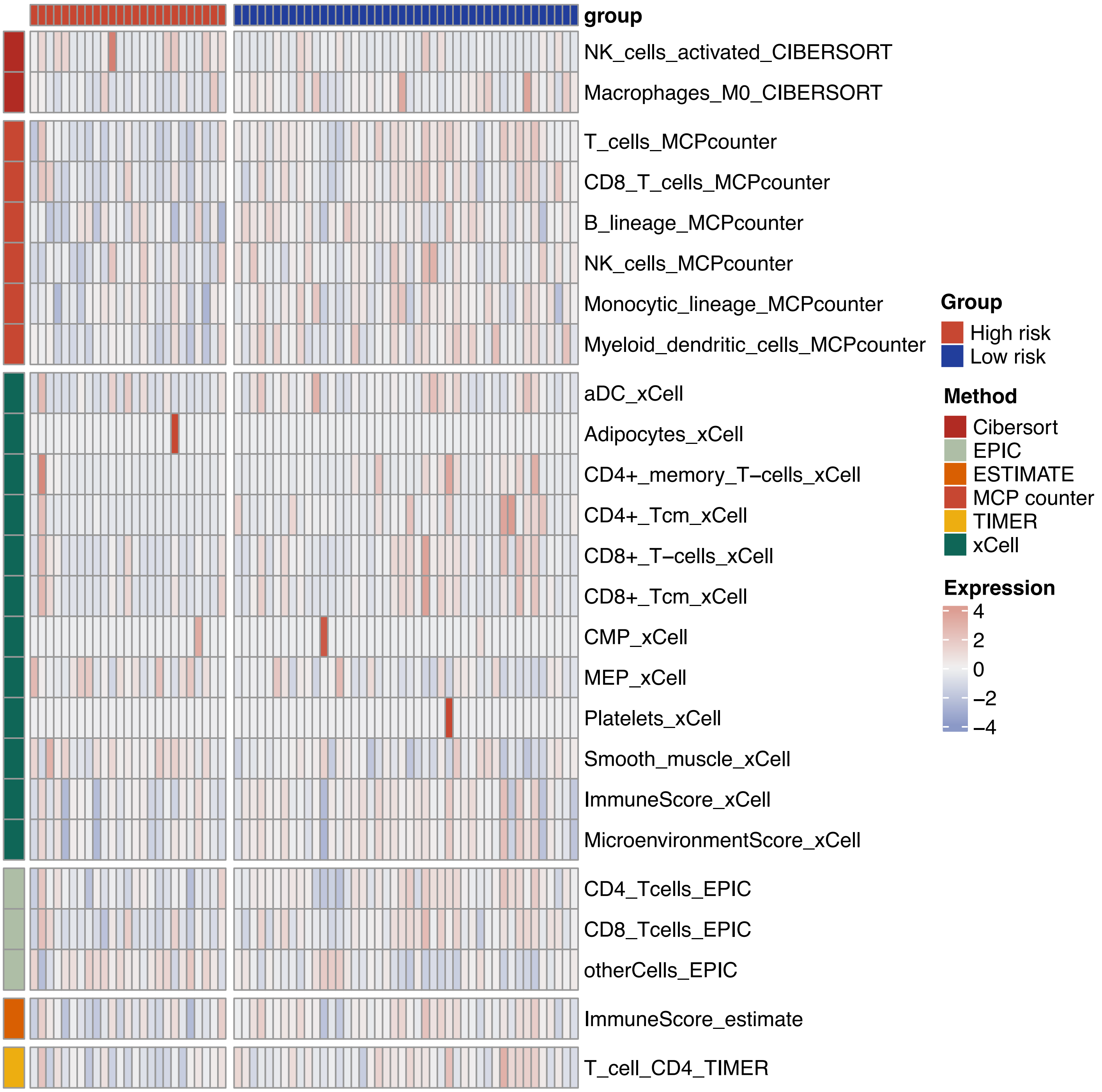

group
NK_cells_activated_CIBERSORT
Macrophages_M0_CIBERSORT
T_cells_MCPcounter
CD8_T_cells_MCPcounter
B_lineage_MCPcounter
NK_cells_MCPcounter
Monocytic_lineage_MCPcounter
Myeloid_dendritic_cells_MCPcounter
aDC_xCell
Adipocytes_xCell
CD4+_memory_T−cells_xCell
CD4+_Tcm_xCell
CD8+_T−cells_xCell
CD8+_Tcm_xCell
CMP_xCell
MEP_xCell
Platelets_xCell
Smooth_muscle_xCell
ImmuneScore_xCell
MicroenvironmentScore_xCell
CD4_Tcells_EPIC
CD8_Tcells_EPIC
otherCells_EPIC
ImmuneScore_estimate
T_cell_CD4_TIMER
Group
High risk
Low risk
Method
Cibersort
EPIC
ESTIMATE
MCP counter
TIMER
xCell
Expression
4
2
0
−2
−4

Figure S8

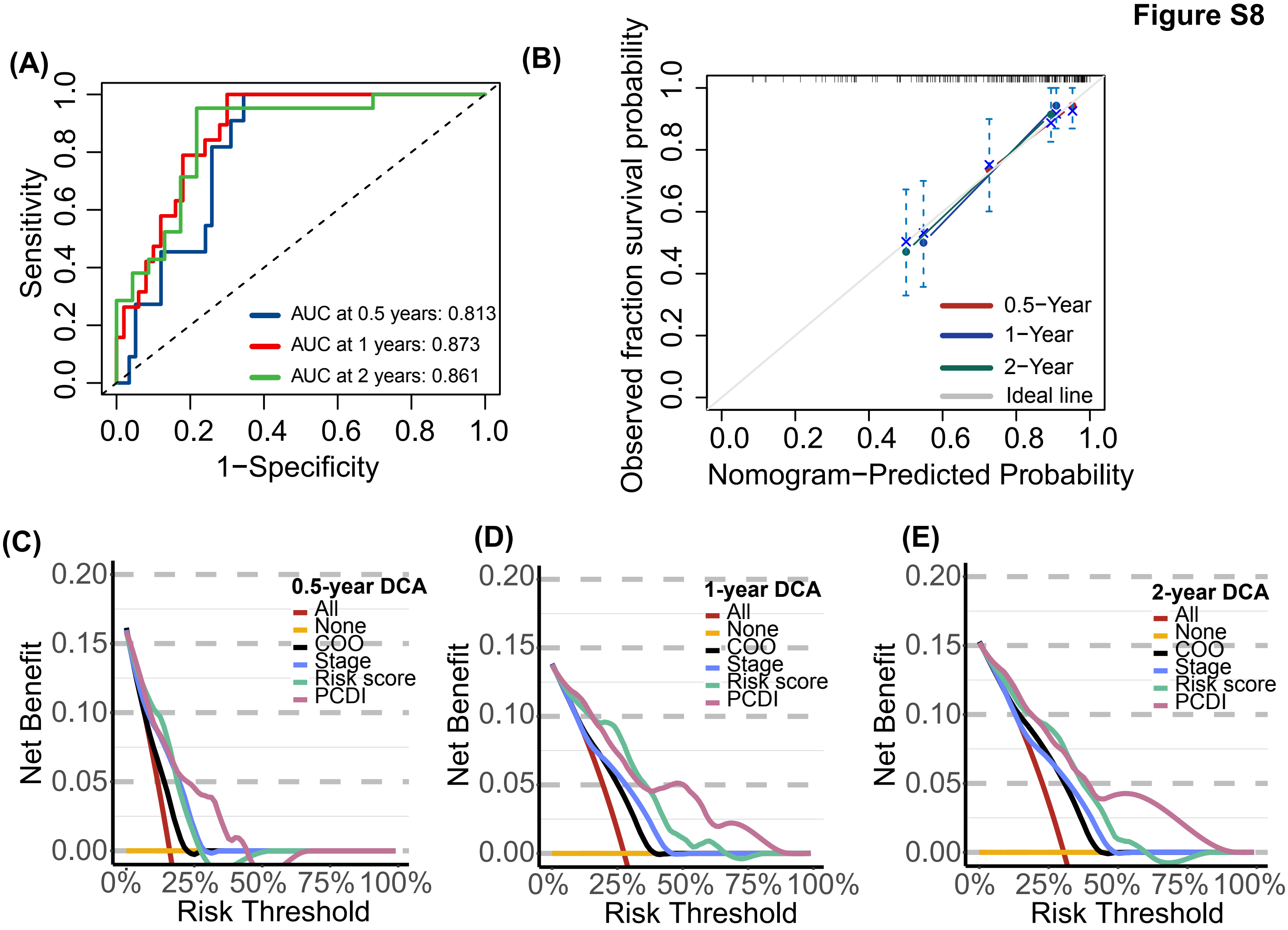

(A)
Sensitivity
1−Specificity
0.0
0.2
0.4
0.6
0.8
1.0
AUC at 0.5 years: 0.813
AUC at 1 years: 0.873
AUC at 2 years: 0.861
(B)
Observed fraction survival probability
Nomogram−Predicted Probability
0.5−Year
1−Year
2−Year
Ideal line
(C)
Net Benefit
Risk Threshold
0.00
0.05
0.10
0.15
0.20
0%
25%
50%
75%
100%
0.5-year DCA
All
None
COO
Stage
Risk score
PCDI
(D)
1-year DCA
(E)
2-year DCA
