# Supplementary material for: Revealing the heterogeneity of treatment resistance in less‐defined subtype diffuse large B cell lymphoma patients by integrating programmed cell death patterns and liquid biopsy
Source: Clin Transl Med. 2024 Dec 27;15(1):e70150. doi: 10.1002/ctm2.70150 (PMC11680560; doi:10.1002/ctm2.70150)
Supplement: Supplementary file 2 — Supporting Information [file CTM2-15-e70150-s002.docx]

Supplementary Material

Supplementary Methods

***Clinical and genetic data***

Eligible patients in public cohort and JSPH cohort met the following criteria: (1) histologically confirmed DLBCL in accordance with the World Health Organization classification; (2) the LymphGen subtype which was predicted using the LymphGen tool was classified as less-defined subtype; (3) underwent first line R-CHOP chemotherapy. The study had access to initial clinical and genetic data, which encompassed demographic details, gender, age, Ann Arbor stage, Eastern Cooperative Oncology Group performance status (ECOG PS), serum lactate dehydrogenase (LDH) levels, bone marrow aspiration and biopsy, International Prognostic Index (IPI), and computed tomography (CT) scans and positron emission tomography/computed tomography (PET/CT) scans, all retrieved from medical records. Tissue and plasma samples from the participants were gathered at the First Affiliated Hospital of Nanjing Medical University. All individuals involved provided their written informed consent for participation in the study. There are no notable statistical disparities observed between these two datasets, confirming their comparability (**Table S1**)

***Liquid biopsy data acquisition***

Targeted next-generation sequencing (NGS) was performed at a Clinical Laboratory Improvement Amendments (CLIA) and College of American Pathologists (CAP)-accredited testing laboratory (Nanjing Geneseeq Technology Inc, Nanjing, China). Sequencing libraries were constructed using the KAPA Hyper Prep Kit (KAPA Biosystems) and sequenced on Illumina HiSeq 4000 using a panel of 475 leukemia- and lymphoma-related genes. The detailed sequence alignment, mutation calling, RNA sequencing, gene expression analysis, tumor clonality and clonal inference were shown in previous published work [1].

Quantitative levels of ctDNA were measured in haploid genome equivalents per milliliter (hGE/mL), determined as the product of total cell-free DNA concentration and the mean allele fraction of somatic mutations, expressed in log scale (log hGE/mL). The formula of ctDNA burden is: $ctDNA burden (log(hGE/ml))=log10(cfDNA \left( ng \right)\times\frac{1000 pg}{1 ng} \times\frac{Haploid Genome Equivalent \left( hGE \right)}{3.3 pg per hGE}\times\frac{Mean Variant Allele Frequency \left( VAF \right)}{Plasma volume \left( ml \right)})$.

Tumor mutation burden (TMB) was counted as the number of all base substitutions and indels per megabyte bases in the coding region of targeted genes, including synonymous alterations.

***Signature generated from machine learning-based integrative approaches***

To establish a consensus on PCD-related genes with high accuracy and stability, we employed a comprehensive by integrating 10 machine-learning algorithms and 70 algorithm combinations. The integrated algorithms encompassed a range of techniques, including random survival forest (RSF), elastic network (Enet), Lasso, Ridge, stepwise Cox, CoxBoost, partial least squares regression forex (plsRcox), supervised principal components (SuperPC), generalized boosted regression modeling (GBM) and survival support vector machine (survival-SVM). The procedure for generating the signatures involved the following steps: (a) Patients from two public databases were divided in an 8:2 ratio based on censor of PFS, resulting in a training dataset of 216 patients and an internal test dataset of 54 patients. The JSPH cohort served as the external test dataset. When comparing the training, internal test, and external test datasets, a few variables exhibited baseline imbalances as depicted in **Table S8**. (b) Survival-related genes were identified by univariate cox analysis in training dataset. (c) The previously identified genes were subjected 70 algorithm combinations to construct predictive models using leave-one-out cross-validation (LOOCV) in the training dataset. (d) All models were further cross-validated using two independent datasets (internal test dataset, and JSPH cohort). (e) For each model, the Harrell’s concordance index (C-index) was calculated across all validation datasets, and the model with the highest average C-index in test dataset was deemed optimal.

***Tumor microenvironment analysis and drug sensitivity prediction***

The expression data of model genes and various immune cell infiltration levels were obtained and calculated using multiple algorithms, including ImmuneCellAI, single-sample Gene Set Enrichment Analysis (ssGSEA) [2], Tumor Immune Estimation Resource (TIMER) [3], Cell-type Identification by Estimating Relative Subpopulations of RNA Transcripts (CIBERSORT) [4], QUANTISEQ [5], Microenvironment Cell Populations-counter (MCPcounter) [6], Xcell [7], and Estimation of Proportion of Immune and Cancer cells (EPIC) [8]. Additionally, we utilized the ‘oncoPredict’ R package to determine the half-maximal inhibitory concentration (IC50) of chemotherapy drugs and predict potential sensitive chemotherapy drugs for high-risk group [9].

***Functional enrichment analysis***

The “limma” R package was utilized to identify different expression genes (DEGs) between different risk groups [10]. The “clusterProfiler” R package was utilized to identify potential Kyoto Encyclopedia of Genes and Genome (KEGG) and Gene Oncology (GO) pathways based on the above-identified DEGs [11, 12]. To compare the distinct biological functions between the high-risk group (high PCDI score) and the low-risk group (low PCDI score), we employed Gene Set Variation Analysis (GSVA) analysis using the “c2.cp.kegg.v7.4.symbols.gmt” database [12].

***Nomogram building and assessment based on the PCDI***

To validate the value of the PCDI as an independent prognostic indicator for other subtype DLBCL patients, both univariate and multivariate Cox regression analyses were conducted. These analyses assessed the significance of the PCDI in combination with relevant clinical parameters. Subsequently, prognostic nomograms were developed based on public databases and the JSPH cohort using the R packages “rms” and “replot” [13]. The performance of nomograms was evaluated through calibration curves, decision curve analysis (DCA), and receiver operating characteristic (ROC) curves.

***Unsupervised clustering of PCD-related model genes***

For unsupervised clustering of the PCD-related model genes, we utilized the “ConsensusClusterPlus” R package [14]. The clustering was performed using agglomerative kindest clustering with a spearman correlation distance metric and 90% of the samples were resampled for 1000 repetitions. To determine the optimal number of clusters, we employed an empirical cumulative distribution function plot. The PFS of other subtype DLBCL patients across different clusters was compared using Kaplan-Meier analysis.

***Statistical analysis***

Differences in PCDI scores and ctDNA concentration between two groups were analyzed using the Unpaired Students' t-test, whereas the one-way analysis of variance test was used to assess the differences among three or more groups. The differences between rates were tested by Chi-square tests or Fisher’s exact tests, if appropriate. PFS was measured from the date of diagnosis to the date of disease progression or death from any cause or the last follow-up. Statistical significance was defined as *P*<0.05. All data analyses were performed using the R software (version 4.3.1), SPSS software (version 20.0) and GraphPad Prism (version 10.0).

**References**

1. Liang, J.H., et al., *Clinical implications of CSF-ctDNA positivity in newly diagnosed diffuse large B cell lymphoma.* Leukemia, 2024. **38**(7): p. 1541-1552.

2. Subramanian, A., et al., *Gene set enrichment analysis: a knowledge-based approach for interpreting genome-wide expression profiles.* Proc Natl Acad Sci U S A, 2005. **102**(43): p. 15545-50.

3. Li, T., et al., *TIMER: A Web Server for Comprehensive Analysis of Tumor-Infiltrating Immune Cells.* Cancer Res, 2017. **77**(21): p. e108-e110.

4. Chen, B., et al., *Profiling Tumor Infiltrating Immune Cells with CIBERSORT.* Methods Mol Biol, 2018. **1711**: p. 243-259.

5. Finotello, F., et al., *Molecular and pharmacological modulators of the tumor immune contexture revealed by deconvolution of RNA-seq data.* Genome Med, 2019. **11**(1): p. 34.

6. Peng, Q., et al., *GLIS1, Correlated with Immune Infiltrates, Is a Potential Prognostic Biomarker in Prostate Cancer.* Int J Mol Sci, 2023. **25**(1).

7. Aran, D., *Cell-Type Enrichment Analysis of Bulk Transcriptomes Using xCell.* Methods Mol Biol, 2020. **2120**: p. 263-276.

8. Racle, J., et al., *Simultaneous enumeration of cancer and immune cell types from bulk tumor gene expression data.* Elife, 2017. **6**.

9. Fu, J., et al., *Large-scale public data reuse to model immunotherapy response and resistance.* Genome Med, 2020. **12**(1): p. 21.

10. Ritchie, M.E., et al., *limma powers differential expression analyses for RNA-sequencing and microarray studies.* Nucleic Acids Res, 2015. **43**(7): p. e47.

11. Wu, T., et al., *clusterProfiler 4.0: A universal enrichment tool for interpreting omics data.* Innovation (Camb), 2021. **2**(3): p. 100141.

12. Kanehisa, M. and S. Goto, *KEGG: kyoto encyclopedia of genes and genomes.* Nucleic Acids Res, 2000. **28**(1): p. 27-30.

13. Blanche, P., J.F. Dartigues, and H. Jacqmin-Gadda, *Estimating and comparing time-dependent areas under receiver operating characteristic curves for censored event times with competing risks.* Stat Med, 2013. **32**(30): p. 5381-97.

14. Wilkerson, M.D. and D.N. Hayes, *ConsensusClusterPlus: a class discovery tool with confidence assessments and item tracking.* Bioinformatics, 2010. **26**(12): p. 1572-3.

Supplementary Tables S1-S8

**Supplementary Table S1:** Clinical data summarized from GSE117556 (N=116) and GSE181063 (N=154).

**Supplementary Table S2:** The list of Programmed cell death related genes (N=2118).

**Supplementary Table S3:** The correlation between risk groups and known and established markers of DLBCL in public cohort and JSPH cohort.

**Supplementary Table S4:** The statistic difference of gene mutation frequencies in plasma sample.

**Supplementary Table S5:** The statistic difference of gene mutation frequencies in tissue sample.

**Supplementary Table S6:** The statistic difference of gene mutation frequencies in patients from public databases.

**Supplementary Table S7:** The statistic difference of immune cell infiltration in patients from the JSPH cohort.

**Supplementary Table S8:** Clinical data summarized from training cohort (N=216), internal test cohort (N=54) and JSPH cohort (N=69).

Supplementary Figure Legends

**Supplementary Figure S1. Kaplan-Meier mortality line was calculated of PFS between patients grouped by expression levels of 8 signature genes from public databases and the JSPH cohort.**

**(A–H)** Kaplan-Meier analysis of PFS for other subtype patients grouped by expression levels of 8 signature genes from public databases.

**(I–P)** Kaplan-Meier analysis of PFS for other subtype patients grouped by expression levels of 8 signature genes from the JSPH cohort.

**Abbr.** PFS, progression-free survival.

**Supplementary Figure S2. Stratified survival analysis was performed using univariate cox analysis to reveal the clinical features of the PFS classifier based on PCDI.**

**(A)** Univariate analysis of the clinical characteristics and PCDI in patients from public databases.

**(B)** Univariate analysis of the clinical characteristics and PCDI in patients from the JSPH cohort.

**Abbr.** PFS, progression-free survival; PCDI, programmed cell death index; COO, cell of origin; IPI, international prognostic index; POD24, progression of disease within 2 years; LDH, lactate dehydrogenase; GCB, germinal center B-cell; ECOG PS, Eastern Cooperative Oncology Group performance status; CR, complete response; PR, partial response; PD, progressive disease; SD, stable disease; MRD, minimal residual disease.

**Supplementary Figure S3. Distribution of PCDI scores between patients grouped by distinct clinical features.**

**(A–I)** Distribution of PCDI scores between patients grouped by distinct clinical features from public databases.

**(J–T)** Distribution of PCDI scores between patients grouped by distinct clinical features from the JSPH cohort.

**Abbr.** PFS, progression-free survival; PCDI, programmed cell death index; COO, cell of origine; IPI, international prognostic index; POD24, progression of disease within 2 years; LDH, lactate dehydrogenase; GCB, germinal center B-cell; ECOG PS, Eastern Cooperative Oncology Group performance status; CR, complete response; PR, partial response; PD, progressive disease; SD, stable disease; MRD, minimal residual disease.

**Supplementary Figure S4. Correlation between the PCDI group and CNS involvement.**

**(A)** Violin plot of the relationship between risk groups and CNS-IPI score.

**(B)** Bar plot showing the distribution of bone marrow, adrenal glands, kidney, breast and CNS involvement between distinct risk groups.

**Abbr.** CNS, central nervous system; IPI, international prognostic index.

**Supplementary Figure S5. Distribution of gene alterations identified in other subtype DLBCL patients with tissue samples.**

**Abbr.** CNV, copy number variant; SV, structural variation.

**Supplementary Figure S6. The underlying biological mechanisms of PCDI groups from the JSPH cohort.**

**(A)** Identification of expression levels of PCDI signature genes between low- and high- risk groups.

**(B)** Heatmap (top 100 upregulated and top 100 downregulated genes) revealed different gene expression patterns between low- and high- risk groups.

**(C) The result of** Kyoto Encyclopedia of Genes and Genome (KEGG) analysis between patients with low- and high- risk.

**(D–E)** Serval important pathways showed by Gene Set Enrichment Analysis (GSEA) between low- and high- risk groups.

Abbr. PCDI, programmed cell death index; KEGG, Kyoto Encyclopedia of Genes and Genome; GSEA, Gene Set Enrichment Analysis.

**Supplementary Figure S7. Immune characteristic between distinct PCDI groups from the JSPH cohort.**

**Abbr.** ssGSEA , single-sample gene set enrichment analysis; TIMER, tumor immune estimation resource; CIBERSORT, cell-type identification by estimating relative subpopulations of RNA transcripts; MCPcounter, microenvironment cell populations-counter; EPIC, estimation of proportion of immune and cancer cells.
